# Identification of Biomarkers for Systemic Distribution of Nanovesicles From *Lactobacillus johnsonii* N6.2

**DOI:** 10.3389/fimmu.2021.723433

**Published:** 2021-08-31

**Authors:** Natalie A. Harrison, Christopher L. Gardner, Danilo R. da Silva, Claudio F. Gonzalez, Graciela L. Lorca

**Affiliations:** Department of Microbiology and Cell Science, Genetics Institute, Institute of Food and Agricultural Sciences, University of Florida, Gainesville, FL, United States

**Keywords:** extracellular vesicles, nanovesicles, probiotic, *Lactobacillus johnsonii* N6.2, IgA, IgG

## Abstract

The ability of bacterial extracellular vesicles (EV) to transport biological molecules has increased the research to determine their potential as therapeutic agents. In this study, *Lactobacillus johnsonii* N6.2-derived nanovesicles (NV) were characterized to identify components that may serve as biomarkers in host-microbe interactions. Comparative proteomic and lipidomic analyses of *L. johnsonii* N6.2 NV and cell membrane (CM) were performed. The lipidomic profiles indicated that both fractions contained similar lipids, however, significant differences were observed in several classes. LC-MS/MS proteomic analysis indicated that NV contained several unique and differentially expressed proteins when compared to the CM. Analysis of Gene Ontology (GO) terms, based on cellular component, showed significant enrichment of proteins in the cytoplasm/intracellular space category for the NV fraction. Based on these results, the proteins T285_RS00825 (named Sdp), Eno3 and LexA were selected for studies of localization and as potential biomarkers for host-microbe interactions. Immunogold staining, followed by scanning and transmission electron microscopy (SEM and TEM, respectively), revealed that Sdp was preferentially localized along the cell wall/membrane, and on NV-like structures surrounding the bacteria. These results were confirmed using immunofluorescence staining in Caco-2 cells incubated with NV. Consequently, we evaluated the potential for NV surface-exposed proteins to generate an immune response in the host. Plasma from individuals administered *L. johnsonii* N6.2 showed that IgA and IgG antibodies were generated against NV and Sdp domains *in vivo*. Altogether, these results show that *L. johnsonii* N6.2 NV have the potential to mediate host interactions through immune modulation.

## Introduction

In recent years, extracellular vesicles have gained increasing attention in the medical and scientific communities due to their ability to mediate cellular communication and transport biological molecules, as well as their potential use as therapeutic agents (1–4). The term extracellular vesicle includes a wide variety of cell-derived membrane structures produced by eukaryotic, prokaryotic and archaeal cells alike (5). In the literature, extracellular vesicles originating from Gram-negative bacteria are generally referred to as outer membrane vesicles (OMVs) (6), while membrane vesicles (MVs) are more commonly associated with Gram-positive bacteria and mycobacteria (7). Herein, we describe nanovesicles (NV) as 20 – 200 nm extracellular vesicles produced by Gram-positive bacteria (8, 9), that transport lipids, proteins, nucleic acids and metabolites between cells to facilitate both local and systemic host-microbe interactions, and can elicit differential effects on recipient cells (5, 10–14).

OMVs have been studied in depth for more than a decade, and their role in pathogenesis, microbial physiology and immune modulation have been well characterized (6). This is in stark contrast to the EVs produced by Gram-positive bacteria, which have only recently been explored. OMVs derived from Gram-negative bacteria have been shown to facilitate biological functions, including communication, competition, biofilm formation, pathogenesis and survival under stress conditions (1). Consequently, OMVs have also been shown to stimulate the innate and adaptive immune response through activation of toll-like receptors (TLRs), NOD-like receptors, and antigen delivery to antigen-presenting cells, which subsequently trigger T-cell and B-cell responses (5).

The mechanism by which NV are formed and released from the cell wall of Gram-positive bacteria remains largely unknown, however, extracellular vesicles have been described in several cell-walled organisms, and their capacity to transport biologically active molecules and elicit local and systemic cellular responses have been documented (9, 11, 15–18). In *Bacillus anthracis*, NV enhance virulence by translocating anthrax toxins to the extracellular space (19), and *Staphylococcus aureus* strain 8325-4 has been shown to enhance virulence by cargoing an α-toxin to host cells (18). In contrast, recent studies in several *Lactobacillus* species have highlighted the potential of NV as beneficial mechanistic effectors. *L. plantarum* WCFS1 up-regulates the host defense genes to enhance the immune response, providing protective effects against vancomycin-resistant enterococci (20). *Lactobacillus rhamnosus* GG-derived NV were reported to inhibit the growth of hepatic cancer cells through apoptotic means, where a significant increase in the *bax/bcl2* expression ratio was observed following incubation of NV with the HepG2 cell line (21). It was also observed that two vaginal *Lactobacillus* species, *Lactobacillus crispatus* BC3 and *Lactobacillus gasseri* BC12, protect human tissues from HIV-1 infection by inhibiting viral attachment of target cells (14).

In this report, we isolate and characterize NV derived from *L. johnsonii* N6.2. We have previously shown that *L. johnsonii* N6.2 mitigates the onset of Type 1 diabetes (T1D) when administered to Biobreeding Diabetes-Prone (BBDP) rats, where administration of the bacteria improved the epithelial barrier by increasing the expression of tight junction proteins and mucus production, while decreasing the intestinal oxidative stress response (22, 23). Further analysis indicated that the prevention of T1D correlated with Th17 cell bias, as well as elevated IL-23 levels in the mesenteric lymph nodes; *in vitro* experiments showed modification of dendritic cells contributed to the Th17 bias (24). The effects of *L. johnsonii* N6.2 were also observed *in vitro* on human intestinal epithelial cells, to determine the TLR signaling pathways and their effects on the innate immune response. Increased expression of TLR7 and TLR9 was observed in response to *L. johnsonii* N6.2 cell-free extracts and pure nucleic acids, suggesting that *L. johnsonii* N6.2 produces bioactive components to aid in the stimulation of the innate immune response (23).

*In vitro, L. johnsonii* N6.2 was also found to produce H_2_O_2_, a strong inhibitor of indoleamine 2,3-dioxygenase (IDO) in the host (25). IDO is the rate limiting step in the kynurenine pathway, where tryptophan is catabolized to produce a variety of immunoregulatory and neuroactive catabolites (26). In BBDP rats administered *L. johnsonii* N6.2, intestinal IDO mRNA levels were lower than the controls. This correlated with the low levels of kynurenine observed in the blood plasma of BBDP rats that received *L. johnsonii* N6.2 (25). In a subsequent clinical trial, healthy adults were administered *L. johnsonii* N6.2 to evaluate the safety, tolerability and general response to this microorganism, where *L. johnsonii* N6.2 was confirmed to be safe for human consumption (27). The administration of *L. johnsonii* N6.2 was also found to promote systemic effects on the circulating leukocytes and metabolites. The tryptophan: kynurenine ratio was significantly different in the treatment group, where increased concentrations of tryptophan in human serum positively correlated with increased *Lactobacillus* cell counts over time. Moreover, significant increases in monocytes and NK cells correlated with the percentage of CD4^+^ T cells, and the serum levels of IgA increased with *L. johnsonii* N6.2 supplementation in human subjects (27).

Taken together, the systemic effects promoted by *L. johnsonii* N6.2 administration in BBDP rats and human studies suggest bioactive components produced by *L. johnsonii* N6.2 may interact with cells at distal locations. We hypothesize *L. johnsonii* N6.2 produces NV that can elicit an immune response in the host. In this study, we isolated and characterized the morphology and molecular composition of NV derived from *L. johnsonii* N6.2. The localization of significantly enriched proteins was determined using SEM and TEM with immunogold labeling, and Sdp domains, SH3b2 and SH3b6, were subsequently identified as biomarkers for host-microbe interactions.

## Materials and Methods

### Bacterial Strains

*Lactobacillus johnsonii* N6.2, isolated from the gut of Biobreeding Diabetes-Resistant rats, was cultivated under anaerobic, static conditions in de Man, Rogosa, Sharpe (MRS) media. As a control, *L. johnsonii* N6.2 was grown under aerobic conditions (100 rpm) until an OD600 of 0.5 was reached. For NV preparation, 500 mL of the MRS media was ultracentrifuged (Beckman) at 175,000 x *g* for 2 h, at 4°C to remove exosomes and extracellular vesicles that may be present in the yeast or beef extract used to make the media, followed by filter-sterilized (0.2 µm). *L. johnsonii* N6.2 was inoculated into the exosome-depleted-MRS at 1% (v/v) and incubated at 37°C to OD600 = 1 (early stationary phase). *Escherichia coli* DH5α and BL21 strains were cultivated in Luria broth supplemented with ampicillin (100 µg/mL) (Sigma-Aldrich, St. Louis, MO, USA) and grown at 37°C for cloning and protein expression, respectively.

### Cell Fractionation, Isolation and Purification of *L. johnsonii* N6.2 NV

500 mL *L. johnsonii* N6.2 cultures were centrifuged at 20,000 x *g* for 20 min, at 4°C and the cell pellet was stored at -80°C until cell fractionation was needed. The supernatant was filter-sterilized (0.2 µm) to ensure the removal of residual bacterial cells. The filtered supernatant was then subjected to ultracentrifugation at 175,000 x *g* for 2 h, at 4°C. The resulting NV pellets were washed twice with PBS using the same parameters in previous ultracentrifugation steps. The NV pellet was stored at -80°C until further use or immediately used for quantification using Nanosight NS300 (Malvern instruments Ltd, Malvern, UK). For fractionation of the *L. johnsonii* N6.2 whole cells, the cell pellet was resuspended in PBS and subjected to cell lysis using a French press. The resulting lysed suspension was centrifuged at 20,000 x *g* for 20 min, at 4°C. The supernatant (cell-free extract/total protein extract, TE) was subjected to ultracentrifugation at 175,000 x *g* for 2 h, at 4°C. The pellet contained the cell membrane (CM) fraction, and the supernatant containing intracellular (IC) proteins was stored at -80°C until further use. The pellet was washed twice with PBS using previous ultracentrifugation parameters and then stored at -80°C.

### Nanoparticle Tracking Analysis

Nanoparticle tracking analysis (NTA) determines size distribution and concentration of particles, utilizing light scattering and Brownian motion. Crude NV pellets were suspended in 400 µL of PBS. The suspension was diluted for optimal measurements and measured using NanoSight NS300 (Malvern instruments Ltd, Malvern, UK) in the University of Florida’s cytometry core at the Interdisciplinary Center for Biotechnology Research. Videos were recorded for 60 s (five times), with the camera level at 15, and analyzed with NTA software 4.3 (Malvern instruments Ltd, Malvern, UK). To calculate the amount of NV per bacterial cell, a ratio of particles/CFU was performed. Calculating a ratio of 1.45 x 10^12^ particles/L over 3.6 x 10^10^ CFU/L estimates that ~40 NV are produced per bacterial cell.

### Untargeted Lipidomics

Similar sized pellets (3 mg) of NV and CM were resuspended and washed in 1 mL of 1% NaCl (w/v). Samples were subsequently ultracentrifuged at 175,000 x *g* for 2 h, at 4°C. The resulting pellet was frozen at -80°C for 2 h. The pellets were introduced to the solvents chloroform, methanol and water in a ratio of 1:2:0.8 (v/v/v), respectively. The mixture stood for 18 h with occasional shaking and was then separated in a separatory funnel with a final chloroform, methanol, and water ratio of 1:1:0.9 (v/v/v). Lipids were extracted from the lower chloroform phase. The chloroform was evaporated in a thermo-heating block at 65°C under a fume hood. Lipids were resuspended in 500 µL of chloroform. Lipid concentration was determined using a colorimetric assay through a sulfuric acid/vanillin/phosphoric acid method. It was standardized against cholesterol dilutions (2mg/mL, 1 mg/mL, 500 µg/mL, 250 µg/mL, 125 µg/mL, 62.5 µg/mL, 31.3 µg/mL, 15.7 µg/mL). Global lipidomic profiling was conducted by the Mass Spectrometry core at the Southeast Center for Integrated Metabolomics at the University of Florida. It was performed on a Thermo Q-Exactive Orbitrap mass spectrometer with Dionex UHPLC and autosampler. All samples were analyzed in positive and negative heated electrospray ionization with a mass resolution of 35,000 at m/z 200 as separate injections. Separation was achieved on an Acquity BEH C18 1.7 µm, 100 x 2.1 mm column with mobile phase A as 60:40 Acetonitrile:10 mM Ammonium formate with 0.1% formic acid in water and mobile phase B as 90:8:2 2-propanol: acetonitrile: 10mM ammonium formate with 0.1% formic acid in water. The flow rate was 500 µL/min with a column temperature of 50°C. 5 µL was injected for negative ions and 3 µL for positive ions.

From both negative and positive ionization lipid determination, the data was analyzed through the normalization of each individual lipid’s peak area over the sum of all the lipids’ peak area detected within one sample of either NV or CM. To determine enriched lipids in each sample, a ratio of the average of NV lipids and an average of CM lipids was conducted. Average of NV over the average of CM lipids, or an average of CM over the average of NV lipids, with a ratio > 1.5 was considered enriched.

### Proteomics

3 mg of NV and CM pellet were resuspended in 1x Laemmli buffer (1M Tris pH 6.8, 100% glycerol, β-mercaptoethanol, DTT, SDS, and 0.005% bromophenol blue). The resulting suspension was incubated in a water bath at 40°C for 30 min. Sodium dodecyl sulfate-polyacrylamide gel electrophoresis (SDS-PAGE) was conducted using the mini-PROTEAN Tetra cell (Bio-Rad, Hercules, CA, USA). Fifteen µL of each sample (Total Extract, Intracellular Extract, Cell Membrane and Nanovesicles) was loaded into the corresponding wells, separating through a homogeneous SDS-PAGE gel (12.5%) for 40 mins at 200 V. The gel was stained using Coomassie Brilliant Blue R-250 (Thermo Fisher Scientific, Waltham, MA, USA).

For proteomic analyses, polyacrylamide gel extracts of three biological replicates from nanovesicle and cell membrane protein samples were sent for mass spectroscopy. 15 µL of each sample was loaded into the corresponding wells, separating through a homogeneous SDS-PAGE gel (12.5%) at 200 V until the sample ran 1.5 cm into the gel. The gel was stained using Coomassie Brilliant Blue R-250 (Thermo Fisher Scientific, Waltham, MA, USA). LC-MS/MS was conducted on these samples by the Proteomics core at the Interdisciplinary Center for Biotechnology Research at the University of Florida. Using zip tip and trypsin in-gel digestion for each sample, each sample was run during a 2 h gradient on the Orbitrap-Fusion instrument.

The LC-MS/MS raw data was analyzed with Scaffold software (version Scaffold_4.8.7; Proteome Software Inc., Portland, OR, USA). Protein identifications were considered positive by recognizing 2 or more peptide hits in at least one of the samples with a peptide threshold of 95% as validated by the Peptide Prophet Algorithm. Blast2Go (version 5.2.5) was used to obtain GO annotations curated from the Scaffold data against the Firmicute database created using Blast2GO with Firmicute data obtained from NCBI. Further analysis on specific proteins identified from the proteomic data included MultAlin, Phobius, TMHMM, and the signal peptide predictor server, SignalP 4.1. Data are available *via* ProteomeXchange with identifier PXD027785.

Simple modular architecture research tool [SMART (28, 29)] was used to identify homologs to protein T285_RS00825. Non-redundant proteins were curated to generate a Newick tree and iTOL database of each protein domain. iTOL was used to generate a phylogenetic tree (30).

### Gene Cloning, Expression and Protein Purification

Standard methods were used for *L. johnsonii* N6.2 genomic DNA isolation (QIAGEN DNeasy Blood and Tissue Kit, Germantown, Maryland, USA), restriction enzyme digestion, agarose gel electrophoresis, ligation and transformation (31, 32). The primers are listed in **Table 1**. The p15TVL (GenBank accession EF456736) vector plasmids were isolated using the QIAprep^®^ Spin Miniprep Kit (QIAGEN, Valencia, CA) and PCR products were purified using QIAquick purification kits (QIAGEN). The p15TVL plasmid clones were transformed into *E. coli* DH5α. Positive selection of transformed bacterial colonies were subjected to colony PCR to amplify the gene insert and verify that the sequence was correct and in frame with the plasmid by sequencing with T7 universal primers (Eton Bioscience). His-tagged fusion proteins were over-expressed in *E. coli* BL1 (DE3). Cells were grown in Luria Broth at 37°C to an optical density of ~0.8 and genes were overexpressed with 0.5 mM isopropyl-tiol-β-D-galactopyranoside (IPTG). After induction with IPTG, the cells were incubated at 17°C for 16 h and harvested by centrifugation at 7,800 x *g* for 20 min. Cells were resuspended in binding buffer (500 mM NaCl, 5% glycerol, 50 nM Tris, 5 mM imidazole, pH 8.0). For protein purification, cells were lysed using a French press and the lysates were centrifuged at 35,000 x *g* for 45 min. The supernatant was applied to a Ni^2+^ affinity column. The column was washed with 200 mL of wash buffer (binding buffer with 25 mM imidazole) and the proteins were eluted with elution buffer (binding buffer with 250 mM imidazole). The purified proteins were dialyzed against 10 mM Tris (pH=8), 2.5% glycerol, 500 mM NaCl and 0.5 mM TCEP, and stored at -80°C (31, 32). Protein concentrations were measured by Bradford assay (Bio-Rad). Bovine serum albumin (BSA; Gold BioTechnology, St. Louis, MO, USA) was used as the standard.

**Table 1:** Primers used for cloning and protein purification.

Primer Names	Locus Tags	Sequences (5’→3’)
SH3b2_Fw	T285_RS00825	TTGTATTTCCAGGGCCCAAGTACAAACACTAACGTAAATA
SH3b2_Rv	T285_RS00825	CAAGCTTCGTCATCACTTTTCTGCTGGTTGACTT
SH3b6_Fw	T285_RS00825	TTGTATTTCCAGGGCGCTAATAAGCCTGTTCGACAAA
SH3b6_Rv	T285_RS00825	CAAGCTTCGTCATCATTATCTGTAAGTTCCCCATGCTT
Enolase_Fw	T285_RS03880	TTGTATTTCCAGGGCATGCTCAAATCAGTTATTGAG
Enolase_Rv	T285_RS03880	CAAGCTTCGTCATCATTAATCTAAGTCAACGTTGTC
LexA_Fw	T285_RS03645	TTGTATTTCCAGGGCATGACTGAACCACATGCAAA
LexA_RV	T285_RS03645	CAAGCTTCGTCATCATTAATCAATATTATTACGATATAGACC

### Polyclonal Antibody Generation

Polyclonal antibodies were generated against purified LexA (T285_RS03645), Enolase (Eno3, T285_RS03880), Sdp_SH3b2 (domain from T285_RS00825) and Sdp_SH3b6 (domain from T285_RS00825) proteins. Approximately 2-3 mg of each protein was provided to Pacific Immunology. For the custom polyclonal antibody production, a 13-week immunization protocol was followed. Two New Zealand White rabbits were immunized against each protein with the first immunization in complete Freund’s adjuvant and boosted with three immunizations with incomplete Freund’s adjuvant. The polyclonal antibodies were purified through affinity column purification (Pacific Immunology Corp.).

### Western Blot Analysis

Western blots were utilized to evaluate the sensitivity and specificity of each antibody. Purified proteins (LexA, Eno3, Sdp_SH3b6, Sdp_SH3b2), NV, and total protein extract (TE) collected from *L. johnsonii* N6.2 under anaerobic and aerobic conditions were loaded at different concentrations (100 ng, 1 µg, 10 µg, and 100 µg). Proteins were separated by SDS-PAGE (10%), at 200 V for 40 mins and transferred at 500 mA for 60 mins onto a polyvinylidene difluoride (PVDF) membrane (Bio-Rad) using a semi-dry blotting unit (Fisher Scientific, Hampton, NH, USA). Membranes were blocked with 5% non-fat dry milk overnight at 4°C. Membranes were washed three times with 0.1% Tween-20 (Fisher Scientific, Hampton, NH, USA) Tris buffered saline (TBS-T). Primary antibodies (anti-LexA, anti-Eno3, anti-Sdp_SH3b2 and anti-Sdp_SH3b6) were incubated in 5% non-fat dry milk overnight at 4°C, at a dilution of 1:1,000. Membranes were washed three times with TBS-T, and the secondary antibody, goat anti-rabbit IgG (H+L) antibody conjugated to horseradish peroxidase (Abcam, Cambridge, MA, USA), was incubated in 5% non-fat dry milk for 1 h at room temperature, at a dilution of 1:2,500. Membranes were washed three times with TBS-T and visualized with ProSignal Femto Solution (Genesee Scientific, San Diego, CA, USA) using the automatic imager FluorChem R (Protein Simple, San Jose, CA, USA).

### Optiprep Density Gradient Purification

Optiprep™ (60% w/v solution of iodixanol in water; Sigma-Aldrich, St. Louis, MO, USA) density gradient ultracentrifugation was performed on the crude pellet of NV resulting from a 1.5 L culture. A working solution of 50% (w/v) iodixanol was made by mixing 5 volumes of 60% (w/v) Optiprep™ in one volume of 6x dilution solution (0.85% NaCl, 60 mM HEPES, pH 7.4). A discontinuous gradient of 50%, 45%, 40%, 35%, 30% and 25% was made by diluting the 50% iodixanol working solution with the 1x dilution solution (DS: 0.85% NaCl, 10 mM HEPES, pH 7.4). Following ultracentrifugation, the crude NV pellet was dissolved in 0.333 mL of the 6x dilution solution and mixed with 1.667 mL of the Optiprep™ solution. The 50% iodixanol solution containing the sample was then overlaid with 2 mL of the 45%, 40%, 35%, 30% and 25% solutions. Ultracentrifugation was performed at 175,000 x g at 4°C for 16 h (SW 41 Ti rotor, Beckman Coulter). From the top, 1 mL fractions were collected and the refraction indices of each fraction was measured using a refractometer with corresponding densities being determined through Optiprep Application Sheet V01. This procedure was carried out twice to perform protein precipitation and to retrieve purified NV pellets.

Each fraction was subjected to Trichloroacetic acid (TCA) protein precipitation, with one volume of 100% TCA to four volumes of sample. The samples were incubated at -20°C for 20 min and centrifuged at 19,000 x *g* for five min. Protein pellets were washed three times with 200 µl of ice cold ethanol and centrifuged at 19,000 x *g* for five min. The pellets were subjected to drying to remove excess ethanol. The samples were immediately resuspended in 100 µl of 4x SDS sample buffer and boiled for 10 min prior to SDS PAGE. The samples were diluted by 1/5 and loaded into the gel at 15 µl per sample (fractions 1-12). Western blots were performed as stated in section 4.8.

Fractions were washed in ~50 mL of DS and combined when necessary. Pellets obtained from the combined fractions 1-2, 3-5, 6-7, and 8-9 were resuspended in 200 µl of PBS, and size and concentration was determined through Nanosight NS300 as stated in section 4.3.

### Electron Microscopy

*L. johnsonii* N6.2 was grown in exosome-free MRS for 24 h. Cells were obtained by centrifugation at 7,000 x *g* for 15 min, at 4°C, washed in exosome-free 0.9% peptone water (w/v) and fixed with 2% glutaraldehyde (v/v) in exosome-free 0.9% peptone water (w/v) at 4°C for transmission electron microscopy (TEM) and Trump’s fixative solution (VWR, USA) for scanning electron microscopy (SEM). All electron microscopy imaging was conducted by the Microscopy core in the University of Florida Interdisciplinary Center for Biotechnology Research.

### Scanning Electron Microscopy

*L. johnsonii* N6.2 cultures were fixed with Trump’s fixative solution (VWR, USA), placed onto 0.2 μm polycarbonate membrane and processed by Pelco BioWave Pro laboratory microwave (Ted Pella, Redding, CA, USA). The samples were washed three times with PBS (pH= 7.2), post-fixed with 1% buffered osmium tetroxide. After the post-fixation step, the samples were washed with distilled water and dehydrated with a graded ethanol series (25%, 50%, 75%, 95%, 100%). The final step was to preserve the surface structures of the samples by drying them in a critical point dryer (Autosamdri-815, Tousimis Research Corp, Rockville MD, USA). The samples were mounted on carbon adhesive tabs on aluminum specimen mounts and Au/Pd sputter coated (DeskV Denton Vacuum, Moorestown, NJ, USA). Digital micrographs were acquired by field-emission scanning electron microscope (SU-5000, Hitachi High Technologies America, Inc. Schaumburg, IL, USA).

### Transmission Electron Microscopy

*L. johnsonii* N6.2 was fixed with 2% glutaraldehyde (v/v) in exosome-free 0.9% peptone water (w/v), at 4°C. Then cells were processed by Pelco BioWave Pro laboratory microwave (Ted Pella, Redding, CA, USA), washed with 0.1M sodium cacodylate buffer without lysine (pH=7.2), fixed with 1% glutaraldehyde/sodium cacodylate buffer containing 500 ppm ruthenium red, washed with sodium cacodylate buffer and post-fixed with buffered 1% osmium tetroxide (500 ppm ruthenium red). The samples were washed again with buffer and distilled water. Fixed cells were dehydrated with graded ethanol series (25%, 50%, 75%, 95%, 100%) followed by anhydrous acetone wash. Then samples were infiltrated in 30%, 50%, 70%, 100% Embed/Araldite epoxy resin containing Z6040 embedding primer (Electron Microscopy Sciences, Hatfield, PA, USA) and cured at 60°C for 48 h. Ultra-thin sections were collected on 100 mesh carbon coated Fromvar copper grids, post-stained with UranyLess and 3% Reynold’s lead citrate for one minute each. Sections were examined with a FEI Tecnai G2 Spirit Twin TEM (FEI Corp., Hillsboro, OR, USA) and digital images were acquired with a Gatan UltraScan 2k x 2k camera and Digital Micrograph software (Gatan Inc., Pleasanton, CA, USA).

The NV were processed for TEM using a glow discharged carbon coated Formvar 400 mesh copper grid floated onto 10 µL droplet of sample for 5 minutes. Excess solution was dabbed off with filter paper and grid was floated on 1% uranyl acetate for 30 seconds. Stain was removed with filter paper, air dried, and examined with FEI Tecnai G2 Spirit Twin TEM (FEI Corp., Hillsboro, OR, USA). Digital images were acquired with Gatan UltraScan 2k x 2k camera and Digital Micrograph software (Gatan, Inc., Pleasaton, CA, USA).

### Cryo-Transmission Electron Microscopy

Three microliter aliquots of the NV were applied to C-flat holey carbon grids (Protochips, Inc.) and vitrified using a Vitrobot™ Mark IV (FEI Co.) operated at 4°C and with ~90% humidity in the control chamber. The vitrified sample was stored under liquid nitrogen and transferred into a Gatan cryo-holder (Model 626/70) for imaging. The sample was examined using a 4k x 4k CCD camera (Gatan, Inc.) on a Tecnai (FEI Co.) G2 F20-TWIN Transmission Electron Microscope operated at a voltage of 200 kV using low dose conditions (~20 e/Å2). Images were recorded with a defocus of approximately -2µm to improve contrast.

### Immunogold Electron Microscopy

#### Immuno-Transmission Electron Microscopy (iTEM)

For immunoelectron microscopy, *L. johnsonii* N6.2 cells were resuspended into primary fixative containing 4% paraformaldehyde and 0.5% glutaraldehyde, in exosome-free 0.9% peptone water (w/v). Fixed cells were processed with the aid of a Pelco BioWave Pro laboratory microwave (Ted, Pella, Redding CA, USA) and SBT digital orbital shaker (Southwest Science, Trenton, NJ, USA). Samples were washed several times in exosome-free 0.9% peptone water (w/v) and encapsulated in buffered 3% agarose. Specimen was water washed and dehydrated in a graded ethanol series, 25%, 50%, 75%, 95%, and 100% followed by 100% anhydrous ethanol. Dehydrated samples were infiltrated in ethanol-lowicryl HM20 resin with Z6040 embedding primer (Electron Microscopy Sciences, Hatfield, PA) at 50% and 100%, and UV cured at -20°C for 24 h. Ultra-thin sections (120 nm) were collected on Formvar-carbon coated 100 mesh nickel grids.

Immunogold labeling for Transmission Electron Microscopy (TEM): Immunogold labeling was performed by exposure of the ultrathin sections at room temperature as follows: sections were treated with NH_4_Cl in high-salt tween (HST) buffer, rinsed with HST, incubated in a blocking solution (1% non-fat dry milk, 0.5% cold water fish skin gelatin, 0.01% Tween-20 in HST), and incubated overnight at 4°C in a moist chamber, in primary antibody (Anti-LexA, Anti-Sdp_SH3b2, Anti-Sdp_SH3b6, and Anti-enolase at 1:200 dilution). Negative control was established by replacing primary antibody with HST. Sections were washed in HST, followed by PBS, and incubated with a goat anti-rabbit IgG (H+L) secondary antibody conjugated to 12 nm colloidal gold in a 1:20 dilution (Jackson Immuno Research Laboratories, West Grove, PA, USA). Sections were further washed in PBS, fixed in Trump’s fixative (Electron Microscopy Sciences, Hatfield, PA), water washed, and post-stained with 2% uranyl acetate and Reynold’s lead citrate.

TEM Imaging: Sections were examined with a FEI Tecnai G2 Spirit Twin TEM (FEI Corp., Hillsboro, OR) operated at 120 kV, and digital images were randomly acquired with a Gatan UltraScan 2k x 2k camera and Digital Micrograph software (Gatan Inc., Pleasanton, CA, USA).

#### Immuno-Scanning Electron Microscopy (iSEM)

Cells were resuspended into primary fixative containing 4% paraformaldehyde and 0.5% glutaraldehyde, in 0.5% peptone water (w/v). Cells (1:50 dilution) were deposited onto poly-L-lysine treated 0.2 μm membrane filters. Filters were incubated onto primary fixative overnight at 4°C in a moist chamber. After fixation, immunogold labeling was performed by exposure of the filters at room temperature as follows: filters were treated with NH4Cl in HST, rinsed with HST, incubated in a blocking solution (1% non-fat dry milk, 0.5% cold water fish skin gelatin, 0.01% Tween-20 in HST), and incubated overnight at 4°C in a moist chamber, in primary antibody (Anti- LexA, Anti-Sdp_SH3b2, Anti-Sdp_SH3b6, and Anti-enolase at 1:200 dilution). Negative control was established by replacing primary antibody with HST. Filters were washed in HST, followed by PBS, and incubated with a goat anti-rabbit IgG (H+L) secondary antibody conjugated to 12 nm colloidal gold (1:20 dilution; Jackson ImmunoResearch Laboratories, West Grove, PA), washed in PBS, fixed in Trump’s fixative (Electron Microscopy Sciences, Hatfield, PA), and water washed. After immunogold labeling, the filters were processed for SEM with the aid of a Pelco BioWave laboratory microwave (Ted, Pella, Redding CA, USA). Filters were dehydrated in a graded ethanol series 25%, 50%, 75%, 95%, 100% and critical point dried (autosamdri-815, Tousimis, Rockville, MD, USA). Filters were mounted on carbon adhesive tabs on aluminum specimen mounts, and carbon coated (Cressington 328/308R, Ted Pella, Redding, CA, USA). Samples were kept under house vacuum until ready to image.

SEM Imaging: Specimens were examined with secondary electrons (SE) and backscatter electrons (BSE), and digital micrographs were randomly acquired with a field-emission SEM (SU-5000, Hitachi High Technologies America, Schaumburg, IL, USA) operated at 5 kV.

Immunogold particles from both iTEM and iSEM were expressed as percentages of each cellular fraction/NV-like structures to the overall amount of immunogold particles detecting each protein. Further analysis was conducted to express the amount of immunogold particles in each cellular fraction/NV-like structure per image. For iTEM, 19 images were counted per protein, whereas 15 images were counted per protein.

### Culture, Immunofluorescence Staining and Confocal Microscopy of Caco-2 Cells

Human epithelial intestinal Caco-2 cells were cultured in 75 cm^2^ flasks (Thermo Fisher Scientific, Denmark) in Eagle’s Minimum Essential Medium (EMEM) (ATCC, Manassas, VA, USA) supplemented with 15% fetal bovine serum (Sigma-Aldrich, St. Louis, MO, USA), 2% of 10,000 units of penicillin, 10 mg of streptomycin and 25 µg of amphotericin B per mL (Sigma-Aldrich, St. Louis, MO, USA) and maintained in an incubator at 37°C, 95% relative humidity, and 5% CO_2_.

To ensure adherence to the coverslips used for imaging, each coverslip was placed into a well within a 6-well plate and treated with 1 mL of 50 µg/mL Poly-D-Lysine hydrobromide solution (Sigma-Aldrich, St. Louis, MO, USA). The coverslips were incubated in the solution for 3 h. After the incubation, the Poly-D-Lysine hydrobromide solution was removed and the treated coverslips were rinsed with 1 mL of sterile water and allowed to dry before introducing the cells.

Caco-2 cells were seeded onto a 6-well plate containing Poly-D-Lysine treated coverslips at 3x10^5^ cells per well, in 2 mL of Caco-2 medium. The cells were incubated for 48 h to reach confluence. Once the cells reached confluence, they were treated with Caco-2 medium containing 1x10^10^ crude vesicles, 1x10^10^ purified vesicles, or the vesicle suspension buffer (PBS) as a control. The cells were incubated for 30 minutes with their respective treatments. After incubation, the cells were rinsed once with 1 mL of 1x PBS and subsequently fixed using 4% paraformaldehyde in 1 mL of PBS for 10 min. Cells were rinsed with PBS containing 10 mM glycine and 0.2% sodium azide (PBS-GSA) between each step. After fixing, the cells were incubated with 1 mL of Hanks’ Balanced Salt Solution (HBSS) containing 1 µg/mL Wheat Germ Agglutinin, and Alexa Fluor 488 conjugate (Thermo Fisher Scientific, Waltham, MA, USA) per well and incubated for 10 minutes to stain the cell membrane. To ensure the Wheat Germ Agglutinin did not stain internal membrane structures, cells were fixed again with 1 mL of 4% paraformaldehyde in PBS for 10 minutes. Cells are permeabilized by treating them with 1 mL of PBS-GSA containing 0.5% Triton X-100 per well for three minutes and blocked with 1 mL of PBS-GSA containing 1% BSA and 0.1% TWEEN 20 per well for 30 minutes. The coverslips were then incubated with PBS-GSA 1% BSA containing 1:100 of the primary anti-Sdp_SH3b2 antibody and incubated for 30 minutes. After removing the primary antibody solution, the cells were rinsed and treated with PBS-GSA 1% BSA containing 1:200 of the secondary goat anti-rabbit IgG (H+L) antibody– Texas Red antibody conjugate (Thermo Fisher Scientific, Waltham, MA, USA). The secondary antibody solution was removed, and the cells were rinsed with PBS. The coverslip was then mounted onto a slide using ProLong Diamond Antifade Mountant with DAPI (Thermo Fisher Scientific, Waltham, MA, USA). The cells were incubated in the dark, at room temperature for 24 h and images were captured using a Zeiss LSM 800 confocal microscope utilizing C-Apochromat 40x/1.20 W Korr objective (Carl Zeiss, Berlin, Germany). Images were captured and processed using the ZEN 2.3 blue edition software (https://www.zeiss.com/microscopy/us/products/microscope-software/zen.html).

### Immunoglobulin ELISA Assay

IgA, IgM and IgG Human enzyme-linked immunoassay (ELISA) kits (Invitrogen, Thermo Fisher Scientific, Waltham, MA, USA) were used following the manufacturer protocols. For each assay, NV and purified proteins (LexA, Eno3, Sdp_SH3b6, Sdp_SH3b2) were coated at 50 µg/ml. Coating with IgA, IgG and IgM were included for the standard calibration curves following the manufacturer recommended concentrations, as well as for determinations of total IgA, IgG and IgM. Plasma samples obtained from a prior human trial where participants were administered a placebo or *L. johnsonii* N6.2 (IRB # 201400370) were tested in triplicate. Antigens were coated on 96-well plates and incubated against the plasma samples of each subject per each timepoint. The primary (anti-IgM, anti-IgA, and anti-IgG) and the secondary (conjugated with horseradish peroxidase) antibodies were used to determine the concentration of specific ACAb to each antigen. Serial dilutions of each plasma sample were used to determine the adequate dilution to detect the specific ACAb. For IgA and IgM, the optimum serum dilution was 1/5 for all antigens, and for total IgA and IgM, dilutions 1/10000 and 1/5000 were optimal, respectively. For IgG, each dilution for each antigen remained variable per subject, however, total IgG had an optimal dilution of 1/50000. The data was expressed as mg/dL and was then normalized by a ratio of specific Ig: total Ig, multiplied by the plasma sample with the highest average total Ig value.

### Statistical Analysis

For both proteomic and lipid analysis, a bivariate analysis was performed using student’s t-test. Blast2Go statistical analysis was determined based on enriched proteins of NV and CM using an enrichment analysis (Fischer’s Exact test) with a cutoff of a false discovery rate (FDR) < 0.05 and *p* < 0.05. For immunoglobulin ELISA analysis, one-way ANOVA, followed by a Tukey post-hoc test was performed using Origin (version 9.7.0.188; 2020; OriginLab Corporation, Northampton, MA, USA). Results were summarized as means ± standard deviation, and significance was defined as *p* < 0.05.

## Results

### *L. johnsonii* N6.2 Produces NV

**Figure 1:**
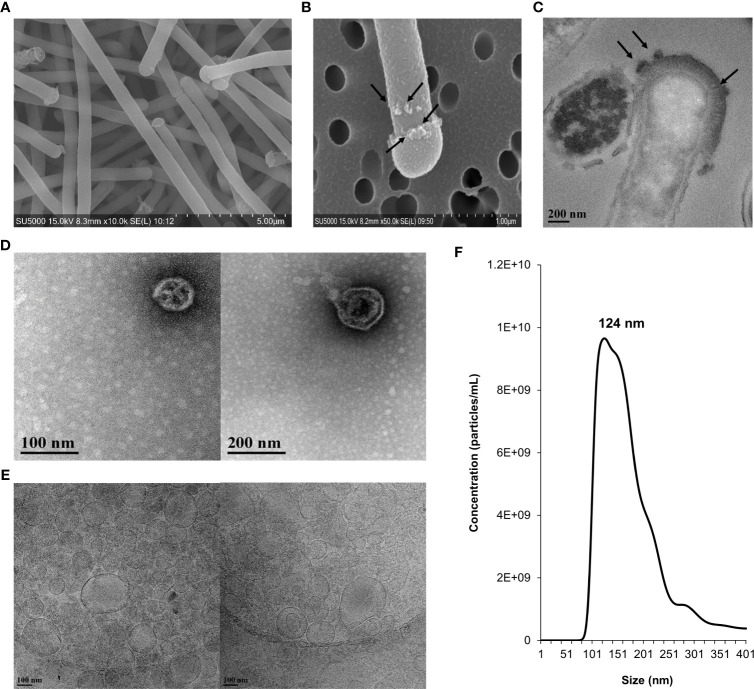
Morphological characterization, size distribution and concentration of NV in *L. johnsonii* N6.2. Whole cells and NV were imaged under electron microscopy and quantified by NanoSight. **(A)** SEM image showing *L. johnsonii* N6.2 cells. **(B)** SEM and **(C)** TEM images depicting the production of NV on the surface of the whole cell and cap structure on the terminal end of the bacterium. Black arrows point to the NV formations. **(D)** TEM and **(E)** Cryo-TEM (right) images of the morphological characteristics of the purified NV. **(F)** Nanoparticle tracking analysis was conducted using Nanosight (NS300).

SEM and TEM were used to confirm that NV structures were produced by *L. johnsonii* N6.2. High magnification SEM images of *L. johnsonii* N6.2 showed large, elongated cells (10.4 ±7 µm) with a spherical structure protruding from the terminal end of the cell, herein referred to as a terminal “cap” (**Figures 1A, B**). The plasma membrane, peptidoglycan layer and proteinaceous S-layer were clearly visualized along the cell periphery using TEM, where the S-layer structures appear to be interrupted in the terminal cap area (**Figure 1B**). NV produced by *L. johnsonii* N6.2 were also visualized using TEM and SEM and were present as spherical protrusions budding off the cell (**Figures 1B, C**). TEM images were also used to examine the highly electro-dense cap structures, which on average were 189.7 ± 74.7 nm wider than the associated bacterial cells, which averaged 558.0 ± 67.1 nm in width.

The size distribution of *L. johnsonii* N6.2 NV was determined by TEM and cryo-TEM imaging, using isolated NV purified from cell-free supernatant (**Figures 1D, E**). The size measurements of NV samples examined by TEM showed an average of 95.8 ± 46.5 nm in diameter. A similar average size of 99.2 ± 48.3 nm was determined from visualization under cryo-TEM. These measurements were consistent with SEM imaging of NV budding from the bacteria cell wall (average size of 96.3 ± 58.6 nm). NTA was conducted to further characterize the size distribution and concentration of NV produced by *L. johnsonii* N6.2. The total yield of NV was 1.45 x 10^12^ particles/L, equivalent to approximately 40 NV per bacterial cell, with a major peak size of 124 nm (**Figure 1F**). The small size differences between the two techniques may be related to the multiple dehydration steps used during TEM. Cell lysis was not observed. To evaluate if the NV are specialized structures budding off the cell, we performed comparative lipidomics and proteomic analyses of the isolated *L. johnsonii* N6.2 cell membranes (CM) and NV.

### *L. johnsonii* N6.2 NV Have a Distinctive Lipid Profile

**Figure 2:**
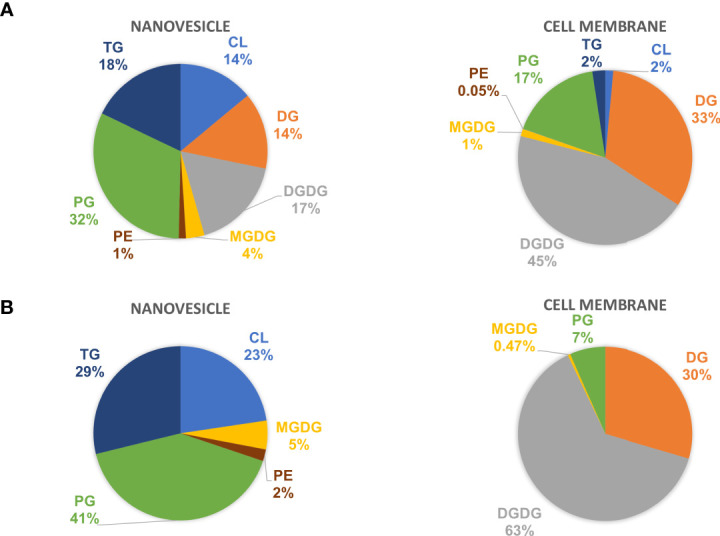
The lipid profiles of NV and CM from *L. johnsonii* N6.2. **(A)** The total lipid composition of NV (left) and CM (right) represented as percentages. **(B)** The enriched lipids found in the NV and CM represented as percentages. PE, glycerophosphoethanolamines; PG, glycerophosphoglycerols; TG, triradylglycerolipids; CL, cardiolipins; MGDG, monogalactosyldiacylglycerol; DGDG, digalactosyldiacylglycerol; DG, diradylglycerolipids.

The lipid composition of NV and CM was analyzed by untargeted global lipidomics using LC-MS/MS using biological triplicates of each fraction. Within the NV and CM total lipid composition, both were found to contain similar lipids, however, the proportions of lipids were significantly different (**Figure 2A**). When compared to the CM, the significant lipids found in NV are glycerophosphoethanolamines (PE; *p* = 0.006), glycerophosphoglycerols (PG; *p* = 0.005), triradylglycerolipids (TG; *p* = 0.02) and cardiolipins (CL; *p* = 0.03). Although not significant, monogalactosyldiacylglycerol (MGDG) lipids were also more abundant in the NV. In contrast, the significant lipids found within the CM are digalactosyldiacylglycerol (DGDG; *p* = 0.0003) and diradylglycerolipids (DG; *p* = 0.02) (**Figure 2A**). An enrichment analysis performed on the lipids in NV (**Table 2**) and CM (**Table 3**) showed a more distinct lipid composition between the two fractions. Lipids were considered enriched when the lipids had a normalized ratio >1.5. The enriched NV lipids were composed of TG (29%), CL (23%), PG (41%), MGDG (5%) and PE (2%), whereas the enriched CM lipids were composed of DG (63%), DGDG (30%), PG (6%) and MGDG (0.47%) (**Figure 2B**). PG lipids were significantly enriched (*p* = 0.01) in the NV when compared to the CM.

**Table 2:** Lipids enriched in the NV of *L. johnsonii* N6.2. The Mean and standard deviation (± SD) was calculated from the relative abundance obtained from each biological triplicate.

Differential Lipids	Molecular Annotation	Ion Mode (+/-)	CM Mean ± SD	NV Mean ± SD	p-value
CL	CL(16:0_18:1_18:1_18:1)	–	0.75 ± 0.26	8.11 ± 1.04	0.025
CL	CL(18:1_18:1_18:1_18:1)	–	1.87 ± 0.02	17.46 ± 2.04	0.029
CL	CL(36:2)(36:2)	+	0.08 ± 0.02	2.03 ± 0.45	0.050
PE	PE(34:2)	+	0.10 ± 0.00	2.67 ± 0.06	0.006
PG	PG(16:0_18:1)	–	2.22 ± 0.12	4.89 ± 0.03	0.007
PG	PG(18:1_18:1)	–	10.18 ± 0.66	17.65 ± 0.97	0.009
PG	PG(17:1_20:2)	+	0.70 ± 0.16	15.43 ± 0.91	0.012
PG	PG(37:3)	+	0.19 ± 0.01	1.66 ± 0.12	0.017
PG	PG(36:2)	+	0.09 ± 0.00	3.05 ± 0.30	0.023
TG	TG(20:3_20:3_20:4)	+	0.06 ± 0.00	2.69 ± 0.03	0.002
TG	TG(16:0_17:1_18:0)	+	0.13 ± 0.00	0.85 ± 0.02	0.006
TG	TG(16:0_18:0_18:0)	+	0.17 ± 0.00	3.45 ± 0.11	0.007
TG	TG(16:0_16:0_18:1)	+	0.32 ± 0.00	3.74 ± 0.19	0.012
TG	TG(16:0_18:0_20:0)	+	0.10 ± 0.00	1.81 ± 0.10	0.013
TG	TG(16:0_18:0_18:1)	+	0.28 ± 0.00	3.62 ± 0.21	0.014
TG	TG(17:0_18:0_18:1)	+	0.08 ± 0.00	0.44 ± 0.03	0.016
TG	TG(16:0_16:0_18:0)	+	0.23 ± 0.01	4.48 ± 0.32	0.017
TG	TG(18:0_18:1_18:1)	+	0.47 ± 0.01	1.96 ± 0.14	0.020
TG	TG(18:1_18:1_18:1)	+	1.09 ± 0.02	3.17 ± 0.21	0.022
TG	TG(16:0_17:1_18:1)	+	0.26 ± 0.01	0.65 ± 0.05	0.024
TG	TG(16:0_18:1_18:1)	+	0.93 ± 0.01	5.24 ± 0.47	0.024
TG	TG(20:3_20:4_20:4)	+	0.07 ± 0.00	3.21 ± 0.41	0.029

**Table 3:** Lipids enriched in the CM of *L. johnsonii* N6.2.

Differential Lipids	Molecular Annotation	Ion Mode (+/-)	CM Mean ± SD	NV Mean ± SD	p-value
DG	DG(16:1_18:1)	+	2.36 ± 0.02	1.90 ± 0.01	0.002
DG	DG(17:1_18:1)	+	3.73 ± 0.27	1.12 ± 0.18	0.006
DG	DG(15:0_18:1)	+	0.50 ± 0.02	0.16 ± 0.01	0.006
DG	DG(18:0_18:1)	+	1.29 ± 0.03	0.71 ± 0.01	0.006
DG	DG(17:0_18:1)	+	2.11 ± 0.13	0.63 ± 0.06	0.007
DG	DG(18:3_19:0)	+	2.75 ± 0.12	0.36 ± 0.04	0.007
DG	DG(18:2_19:0)	+	31.48 ± 1.86	5.10 ± 0.06	0.016
DG	DG(18:1_18:1)	+	19.27 ± 0.48	16.79 ± 0.28	0.020
DGDG	DGDG(18:1_20:1)	–	1.10 ± 0.03	0.04 ± 0.02	0.001
DGDG	DGDG(17:1_18:1)	–	13.47 ± 0.42	2.49 ± 0.32	0.001
DGDG	DGDG(17:0_18:1)	–	13.82 ± 0.38	5.46 ± 0.27	0.001
DGDG	DGDG(15:0_18:1)	–	1.59 ± 0.05	0.43 ± 0.07	0.003
DGDG	DGDG(16:0_18:1)	–	6.60 ± 0.21	3.85 ± 0.22	0.003
DGDG	DGDG(16:1_18:1)	–	5.62 ± 0.15	2.97 ± 0.07	0.004
DGDG	DGDG(18:2_19:0)	+	6.69 ± 0.47	0.62 ± 0.29	0.004
DGDG	DGDG(18:0_18:1)	+	0.22 ± 0.02	0.05 ± 0.02	0.005
DGDG	DGDG(17:0_18:1)	+	3.29 ± 0.25	0.85 ± 0.26	0.005
DGDG	DGDG(17:1_18:1)	+	4.03 ± 0.29	0.52 ± 0.15	0.006
DGDG	DGDG(14:0_18:1)	–	1.37 ± 0.02	0.35 ± 0.06	0.007
DGDG	DGDG(18:1_18:2)	–	1.07 ± 0.01	0.16 ± 0.05	0.011
DGDG	DGDG(18:1_18:1)	–	20.20 ± 0.74	12.51 ± 0.22	0.015
DGDG	DGDG(15:0_18:1)	+	0.56 ± 0.05	0.14 ± 0.01	0.019
DGDG	DGDG(18:1_18:1)	+	5.00 ± 0.39	2.33 ± 0.65	0.028
DGDG	DGDG(18:1_20:1)	+	0.22 ± 0.03	0.01 ± 0.01	0.029
DGDG	DGDG(16:0_18:1)	+	1.73 ± 0.14	0.83 ± 0.24	0.034
DGDG	DGDG(10:0_18:1)	–	0.41 ± 0.01	0.10 ± 0.05	0.037
MGDG	MGDG(18:2_19:0)	+	0.68 ± 0.03	0.34 ± 0.03	0.004
PG	PG(17:1_18:1)	–	6.26 ± 0.33	3.45 ± 0.16	0.011
PG	PG(37:2)	+	3.03 ± 0.53	1.24 ± 0.41	0.035
TG	TG(17:1_18:0_18:1)	+	0.62 ± 0.00	0.45 ± 0.00	0.001

The Mean and standard deviation (± SD) was calculated from the relative abundance obtained from each biological triplicate.

### *L. johnsonii* N6.2 NV Have a Distinctive Proteome

SDS-PAGE was carried out to evaluate the protein profiles of NV, CM, intracellular fraction (IC) and total protein extract (TE) from *L. johnsonii* N6.2 (**Figure S1**). The NV protein profile was found to be significantly different among the four fractions analyzed, while the patterns obtained from the IC and CM fractions were both present in the TE profile. Proteomic analysis was subsequently performed using liquid chromatography-tandem mass spectroscopy (LC-MS/MS) with proteins extracted from the NV and CM fractions. Proteomic analysis indicated that NV contained several unique and differentially expressed proteins when compared to the CM (**Table 4**). 708 protein clusters were identified between the CM and NV triplicates where 524 of these protein clusters were identified in the CM and 366 identified in the NV.

**Table 4:** Enriched protein composition found within the NV as compared to the CM of *L. johnsonii* N6.2.

Locus Tag	Annotation	NV Mean ± SD	CM Mean ± SD	p-value
T285_RS00825	Lysin	35.35 ± 10.29	4.48 ± 0.002	0.018
T285_RS01325	Dipeptidase	33.34 ± 12.02	4.08 ± 4.32	0.020
T285_RS01720	2,3-bisphosphate-dependent phosphoglycerate mutase	28.50 ± 6.44	1.70 ± 1.19	0.008
T285_RS02260	Alanine–tRNA ligase	25.33 ± 11.08	3.37 ± 2.26	0.035
T285_RS02345	Membrane protein/penicillin-binding protein	86.55 ± 14.30	19.40 ± 8.81	0.002
T285_RS02490	Arginine–tRNA ligase	15.62 ± 4.95	2.61 ± 1.58	0.017
T285_RS03880	Enolase 3	355.64 ± 40.61	45.98 ± 19.86	<0.001
T285_RS03940	L-lactate dehydrogenase	76.53 ± 9.23	4.61 ± 2.46	0.002
T285_RS04000	GTP pyrophosphokinase	21.60 ± 3.22	7.31 ± 5.10	0.016
T285_RS04015	Aspartate–tRNA ligase	19.19 ± 9.56	1.17 ± 0.38	0.041
T285_RS04110	Sigma factor SigA	10.58 ± 0.05	5.32 ± 2.83	0.042
T285_RS04625	Penicillin-binding protein 1A	40.65 ± 11.74	21.23 ± 2.95	0.048
T285_RS05135	Hu family DNA-binding protein	15.67 ± 3.26	5.10 ± 1.31	0.009
T285_RS05565	GTP-binding protein TypA	26.82 ± 0.66	10.97 ± 7.87	0.036
T285_RS05640	Isoleucine–tRNA ligase	17.34 ± 5.89	0.75 ± 0.0004	0.020
T285_RS05935	Glucose-6-phosphate isomerase	25.94 ± 12.35	1.12 ± 0.53	0.037
T285_RS06185	Triosephosphate isomerase	18.36 ± 8.51	3.36 ± 0.53	0.046
T285_RS06195	Glyceraldehyde-3-phosphate dehydrogenase	98.35 ± 15.73	15.67 ± 2.69	0.005
T285_RS06765	Threonine–tRNA ligase	38.42 ± 6.29	4.48 ± 0.003	0.006
T285_RS07040	Aspartyl/glutamyl-tRNA(Asn/Gln) amidotransferase subunit B	27.22 ± 9.02	7.38 ± 1.44	0.030
T285_RS07350	Bifunctional acetaldehyde-CoA/alcohol dehydrogenase	40.53 ± 4.78	15.62 ± 11.33	0.024
T285_RS08820	6-phosphogluconate dehydrogenase, decarboxylating	15.50 ± 4.77	1.49 ± 1.05	0.015
T285_RS08830	tRNA uridine 5-carboxymethylaminomethyl modification enzyme (MnmG)	10.39 ± 0.32	4.04 ± 1.06	0.003

The Mean and standard deviation (± SD) was calculated from the relative abundance obtained from each biological triplicate.

**Figure 3:**
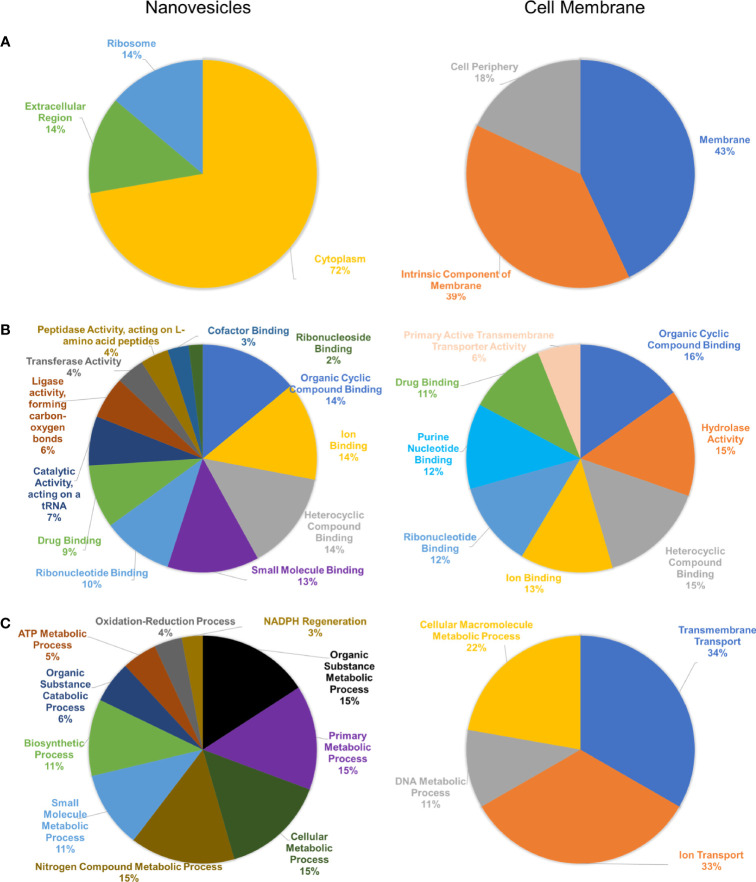
Bioinformatic analysis of protein composition of NV and CM from *L. johnsonii* N6.2. **(A)** Cellular component GO terms of NV (left) and CM (right). **(B)** Molecular function GO terms of NV (left) and CM (right). **(C)** Biological processes GO terms of NV (left) and CM (right).

Blast2Go analysis was conducted against the protein FASTA dataset provided through LC-MS/MS. These analyses included an enrichment Fisher’s Exact test to determine the statistical significance of enriched proteins from NV and CM fractions, as well as a comparison of the protein composition of the NV and CM under the three Gene Ontology (GO) term categories: cellular component, molecular function and biological processes. Analysis of GO terms based on cellular component (**Figure 3A**), indicates the enriched proteins of NV that were significantly (*p* < 0.002) associated with GO terms of cytoplasmic/intracellular proteins. Analysis of the molecular function GO terms (**Figure 3B**) showed the enriched proteins of NV are ligase activity forming carbon-oxygen bonds (*p* = 1.93 x 10^-5^), and catalytic activity acting on a tRNA (*p* = 2.59 x 10^-5^). The enriched proteins of the CM were significant in the GO term primary active transmembrane transporter activity (*p* = 1.07 x 10^-4^). According to the GO term biological processes (**Figure 3C**), the proteins found in the CM were significant in ion transport (*p* = 1.21 x 10^-4^) and transmembrane transport (*p* = 5.69 x 10^-6^). The proteins found in the NV were significant in NADPH regeneration (*p* = 1.86 x 10^-5^), oxidation-reduction process (*p* = 9.45 x 10^-4^), ATP metabolic process (*p* = 2.94 x 10^-8^), organic substance catabolic process (*p* = 2.41 x 10^-6^), biosynthetic process (*p* = 4.76 x 10^5^), small molecule metabolic process (*p* = 9.91 x 10^-8^), nitrogen compound metabolic process (*p* = 9.89 x 10^-6^), cellular metabolic process (*p* = 3.26 x 10^-5^), primary metabolic process (*p* = 7.05 x 10^-6^), and organic substance metabolic process (*p* = 3.72 x 10^-6^). Cellular component localization of proteins enriched in the CM did not reach statistical significance.

Several proteins were found at equally high concentrations in NV and CM, including a sugar ABC transporter substrate-binding protein, enolase 1, 30S ribosomal proteins (S2, S3 and S5), ABC transporter substrate-binding protein (periplasmic binding protein type 2 superfamily), foldase protein PrsA, ABC transporter substrate-binding protein family 5, extracellular solute-binding protein (T285_RS02130) and 50S ribosomal protein L1. Proteins unique to NV are adenylate kinase, aminopeptidase C, cysteine-tRNA ligase, acetate kinase, leucine-tRNA ligase, phosphoketolase, transcription termination/antitermination protein NusA, ATP-dependent 6-phosphofructokinase, dTDP-glucose 4,6-dehydratase, levansucrase, UDP-glucose 4-epimerase, phosphoenolpyruvate-protein phophotransferase, peptidase, surface protein (aggregation promoting factor; T285_RS07210), hypothetical protein (aggregation promoting protein; T285_RS07215), pyroglutamyl-peptidase I and adhesion (**Table 5**). In summary, the enriched proteins identified in NV mostly consist of cytoplasmic proteins involved in glycolysis and protein synthesis. These results confirm that NV are the product of active budding of the cell wall rather than the result of cell lysis.

**Table 5:** Proteins uniquely identified in the NV of *L. johnsonii* N6.2.

Locus Tag	Annotation	Mean ± SD
T285_RS01605	Adenylate kinase	7.79 ± 7.58
T285_RS01810	Aminopeptidase C	19.30 ± 14.44
T285_RS01835	Cysteine–tRNA ligase	1.89 ± 0.71
T285_RS02315	Acetate kinase	29.16 ± 3.69
T285_RS02460	Leucine–tRNA ligase	8.37 ± 6.92
T285_RS03045	Phosphoketolase	36.09 ± 10.16
T285_RS03745	Transcription termination/antitermination protein NusA	5.23 ± 5.26
T285_RS05200	ATP-dependent 6-phosphofructokinase	18.99 ± 7.35
T285_RS05350	dTDP-glucose 4,6-dehydratase	4.48 ± 2.27
T285_RS05980	Levansucrase	6.50 ± 1.36
T285_RS06305	UDP-glucose 4-epimerase (GalE)	11.50 ± 8.38
T285_RS06485	Phosphoenolpyruvate-protein phosphotransferase	18.81 ± 13.66
T285_RS06950	Peptidase	35.34 ± 8.15
T285_RS07210	Surface protein, aggregation promoting factor	23.02 ± 5.46
T285_RS07215	Hypothetical protein (aggregation promoting protein)	8.72 ± 3.21
T285_RS08570	Pyroglutamyl-peptidase I	8.47 ± 4.30
T285_RS08930	Adhesin	43.15 ± 39.12

The Mean and standard deviation (± SD) was calculated from the relative abundance obtained from each biological triplicate.

### Proteins Identified From NV and CM as Potential Biomarkers

Several proteins were selected for antibody production to evaluate their use as potential biomarkers for NV. For this experiment, Eno3 was selected since it is found at high concentrations and represents the category of metabolism, while T285_RS00825, a protein annotated as a SH3b domain-containing protein (herein named Sdp), was selected since it is a membrane bound protein. Both proteins were significantly enriched in the NV fraction (*p* = 0.02 and *p* = 0.002, respectively). LexA (23 kDa) is a cytoplasmic transcriptional regulator that represses genes involved in the SOS response and is associated with oxidative stress responses (33). LexA was selected as an internal control since it was not present in the NV or CM fractions.

Sdp contains a glycosyl hydrolase family 25 (GH25) domain near its N-terminal, and six tandem repeats containing src-homology-3 (SH3b) domains (**Figure S2A**). Sdp has a predicted MW of 102 kDa, with a predicted signal peptide at the N-terminal region of the protein, prior to the GH25 domain, with a cleavage site between residues 38A and 39A. Analysis using transmembrane prediction servers (Phobius and TMHMM) suggest the protein is located outside the cell (or non-cytoplasmic), with TMHMM indicating a 40% probability that the N-terminal is located in the membrane. Homologs of Sdp in other *Lactobacillus species* contain two or less SH3b domains (**Figure S3**), with the exception of *L. taiwanensis* strains which have four to six SH3b domains, and *L. gasseri* 224-1 which has six SH3b domains. We hypothesize *L. johnsonii* N6.2 Sdp is inserted in the membrane of the NV, and the SH3b domains are located outside of the NV. Sequence alignments performed with each of the SH3b domains (numbered 1 through 6 from N- to C- terminus, respectively), showed that SH3b2 has higher identity to the other domains within Sdp, while SH3b6 showed more sequence divergence (**Figure S2B**). Based on these results, the SH3b2 (named Sdp_SH3b2) and SH3b6 (named Sdp_SH3b6) domains were selected for protein purification and subsequently used to determine localization of Sdp in NV and *L. johnsonii* N6.2 whole cells.

Enolase is a metalloenzyme that is primarily involved in the conversion of 2-phosphoglycerate to phosphoenolpyruvate in the glycolysis pathway, however, a role in bacterial adhesion binding to the extracellular matrix, mucin and other proteins has also been described (34–37). Similar reports have shown enolase to be a cell surface protein capable of binding human plasminogen, laminin, or fibronectin (38). Three enolases were identified in the genome of *L. johnsonii* N6.2. Enolase three (Eno3) shares 72.88% identity with enolase 2, and 56.04% identity with enolase 1. Eno3 (47 kDa) was present in the NV and CM fractions and was also significantly enriched in NV (*p* = 8 x 10^-4^).

The selected genes or domains (*sdp_SH3b2, sdp_SH3b6, lexA* and *eno3*) were cloned to fuse a His-tag and subsequently purified using Ni-NTA agarose. The yields obtained were 576 mg/L, 537 mg/L, 6760 mg/L and 2960 mg/L for Sdp_SH3b2, Sdp_SH3b6, LexA and Eno3, respectively (**Figure S2C**). The purified proteins were used for the generation of polyclonal antibodies. The detection limit of each antibody was determined using purified protein, where the detection limit for anti-sdp_SH3b2 and anti-sdp_SH3b6 were found to be 1 ng and 100 ng, respectively. The detection limit for anti-Eno3 and anti-LexA were both 1 ng of purified protein (Eno3 and LexA, respectively).

**Figure 4:**
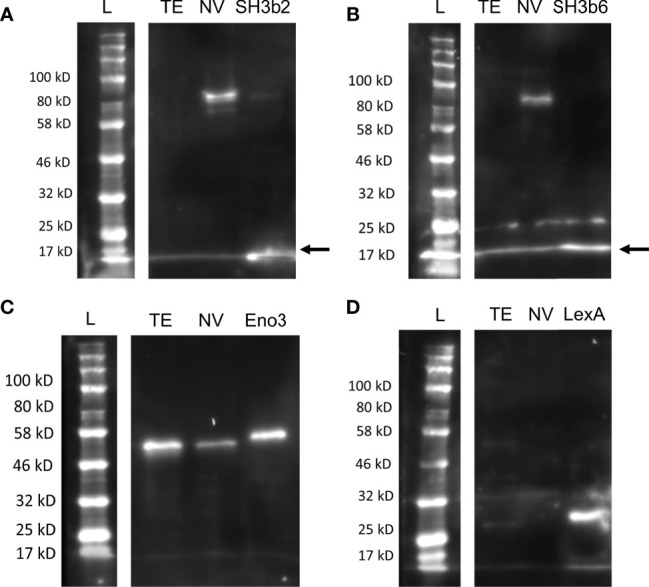
Distribution of Sdp_SH3b2, Sdp_SH3b6, Eno3 and LexA in cell fractions. Representative Western blots are shown using anti-Sdp_SH3b2 **(A)**, anti-Sdp_SH3b6 **(B)**, anti-Enolase **(C)**, and anti-LexA **(D)** against *L. johnsonii* N6.2 total protein extract (TE) and NV. The corresponding purified protein was used as a positive control in each gel. The predicted size of the purified Sdp_SH3b2 and Sdp_SH3b6 domains is ~15 kDa. When present in NV, the predicted size for the detection of Sdp_SH3b2 and Sdp_SH3b6 is ~100 kDa (Sdp). The predicted MW of LexA and Eno3 is 27 kDa and 47 kDa, respectively. Arrows are indicating the band of the pure protein.

Western blots were conducted with NV and *L. johnsonii* N6.2 total cell extract (TE) to further examine the specificity of antibodies generated against Sdp_SH3b2, Sdp_SH3b6, Eno3 and LexA, and to determine the presence or absence of these proteins within the NV and TE fractions (**Figure 4**). The native Sdp (~100 kDa) was detected in the NV fraction using anti-Sdp_SH3b2 (**Figure 4A**) and anti-Sdp_SH3b6 (**Figure 4B**), however, neither domain was detected in the TE fraction. Eno3 was detected on both cellular fractions (**Figure 4C**), in agreement with the high abundancy of this protein observed in the proteomic analyses. LexA was only detected against the purified protein and was not observed in the NV or TE fraction (**Figure 4D**). The reactivity of the anti-lexA antibody was confirmed in *L. johnsonii* N6.2 total cell extract (TE) grown in aerobic conditions (**Figure S4**).

To confirm that each of the identified proteins was a component of the NV cargo and not an artifact of cellular debris or protein aggregation from the extraction process, Optiprep™ density gradient was performed on the crude NV pellet. Based on the refractive index, the density of the purified NV was determined to be 1.16 – 1.26 g/mL. These results are in agreement with a recent report in *L. plantarum* (39). Following density gradient centrifugation using Optiprep™, the presence of NV-derived proteins within each fraction was evaluated by western blot using anti-Sdp_SH3b2 (**Figure S5A**). Fractions 3 through 9 had prominent bands detected in the western blot while no signal was observed in fractions 10, 11 or 12 using anti-Sdp_SH3b2 (**Figure S5A**). Fraction 12 is where free protein aggregates are collected.

The association of Sdp with NVs was confirmed by further NV purification from the different fractions. Due to the concentration obtained, the fractions were pooled and subjected to ultracentrifugation to obtain F1 (fractions 1 and 2), F2 (fractions 3, 4 and 5), F3 (fractions 6 and 7), F4 (fractions 8 and 9) and F5 (fractions 10, 11 and 12). Following the ultracentrifugation step, F1 resulted in a very small pellet, fractions F2, F3 and F4 resulted in similar sized pellets, and no pellet was obtained from fraction F5. The NV size and concentration was determined for fractions F1, F2, F3 and F4, and compared to the crude NV pellet using NS300 (**Figure S5B**). The concentration of F1 was 5.81x10^9^ particles/L, F2 was 9.67 x 10^10^ particles/L, F3 was 1.11 x 10^11^ particles/L and F4 was 1.67 x 10^11^ particles/L, and the crude NV had a concentration of 1.45 x 10^12^ particles/L. The main peaks observed with each fraction corresponded to particle sizes ranging from 110 nm to 136 nm, consistent with the average particle size (124 nm) observed in the crude NV pellet (**Figure S5B**). Western blots were subsequently conducted against Sdp_SH3b2, Sdp_SH3b6, Eno3 and LexA to further examine the distribution of the selected proteins within each purified NV fraction. When compared to the crude NV, similar banding of the Sdp_SH3b2, Sdp_SH3b6 and Eno3 was visualized in the purified NV fractions (**Figure S5C**). The control LexA was not visualized in any of the fractions or crude NV. Based on these results, it was concluded that our approach to using exosome-free MRS media for NV preparation is sufficient to obtain a crude extract of NV with a similar size and protein composition to the purified NV.

### Protein Localization Visualized Using Immunogold and Immunofluorescence Staining

**Figure 5:**
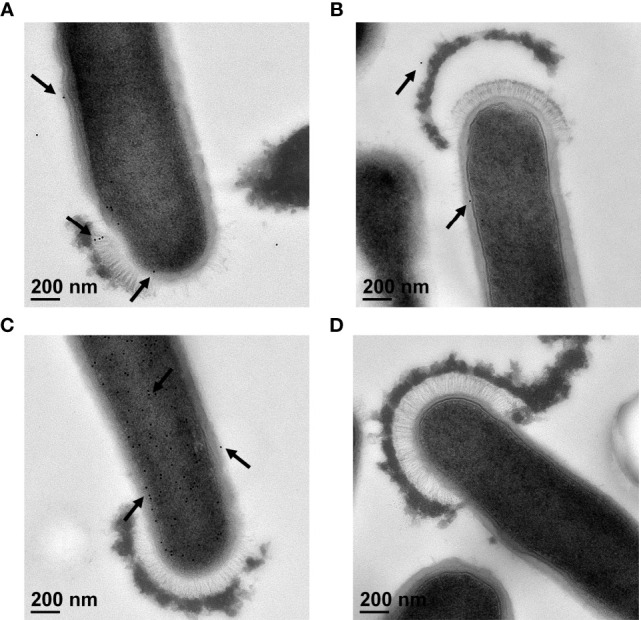
Immunogold staining of *L. johnsonii* N6.2 whole cells. Representative TEM images of immunogold staining of anti-Sdp_SH3b2 **(A)**, anti-Sdp_SH3b6 **(B)**, anti-Eno3 **(C)** and anti-LexA **(D)**. Black arrows indicate location of 12 nm gold particles that appear as black round dots.

Antibodies generated against Sdp_SH3b2, Sdp_SH3b6, Eno3 and LexA were used to confirm the localization of each protein through immunogold staining and visualization under TEM. Anti-LexA and secondary antibody alone were used as a negative control. Representative TEM images are shown in **Figure 5** and a summary of the quantitative TEM analyses are shown in **Table S1**. Each image (n=19 per antibody) was counted in respect to the number of total immunogold bacterial particles determined on each cell, and free particles located on the surrounding grid. Bacterial particles were further analyzed based on their association with bacterial structures, such as the cell wall, cell membrane, cytoplasm, and cap-like structures.

Using anti-Sdp_SH3b2, 94% of the gold particles (n = 907) were associated with bacterial structures **(** **Figure 5A**). Of those, 3% of the particles were associated with the cell wall, 25% with the cell membrane, 71% were detected in the cytoplasm, and 1% of the particles were located in cap-like structures (**Table S1**). Anti-Sdp_SH3b6 resulted in a lower number of immunogold particles detected overall (n = 439), as well as associated with bacterial structures (48%) (**Figure 5B** and **Table S1**). Anti-Sdp_SH3b6 particles bound to bacterial structures were further divided into the number associated with the cell wall (13%), cell membrane (30%), cytoplasm (55%), and the cap-like structures (1%). We speculate that the decreased signal obtained may be due to gold particles being released from the NV during washing steps. Only anti-Sdp_SH3b2 and anti-Sdp_SH3b6 were found to show immunogold particles localized to the cap-like structure, at 6 and 3 particles, respectively.

In contrast, 99% of the gold particles were localized in bacterial structures using anti-Eno3 (**Figure 5C**; n = 7687). Of the anti-Eno3 bacteria-associated particles, 4% were located on the cell wall, 16% at the cell membrane, and 80% were found within the cytoplasm. The statistical analyses showed that this protein was significantly (*p* < 0.001) associated with the cytoplasmic fraction when compared to the cell wall and cell membrane. Similarly, Eno3 showed a significant localization in the cytoplasm compared to that of SH3b2 (*p* < 0.001) and SH3b6 (*p* < 0.001). As expected, using anti-LexA or the secondary antibody controls resulted in the detection of very low numbers of gold particles (**Figure 5D** and **Table S1**).

**Figure 6:**
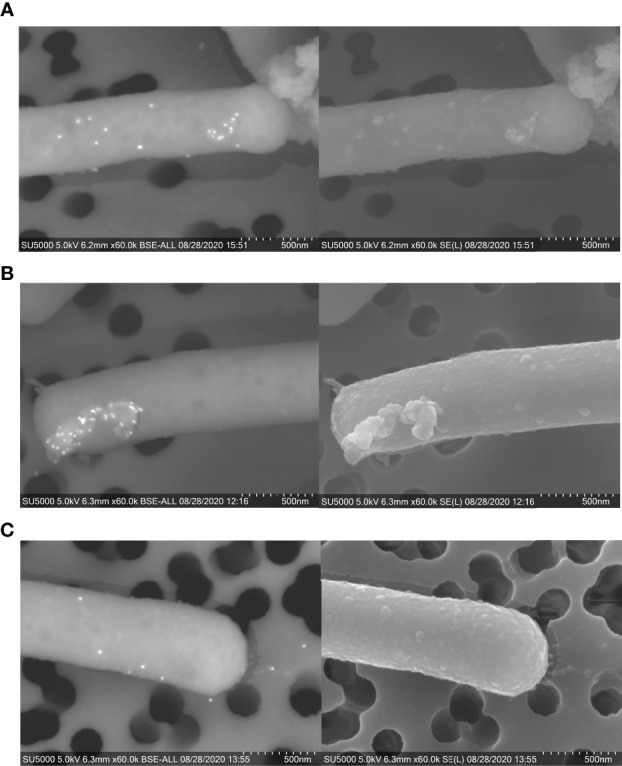
Immunogold staining of *L. johnsonii* N6.2 whole cells. SEM [BSE (left) and SE (right)] images of immunogold staining of Sdp_SH3b2 **(A)**, Sdp_SH3b6 **(B)**, and Eno3 **(C)**. On BSE images, 12 mm gold particles appear as fluorescent dots.

To evaluate the presence of these proteins on the surface of NV and whole *L. johnsonii* N6.2 cells, immunogold staining was performed through SEM. Whole bacteria cells were used for imaging due to the size limitations in working with purified NV. Gold particles conjugated to anti-Sdp_SH3b2, anti-Sdp_SH3b6 and anti-Eno3 were present on the cell surface (**Figure 6**), where the highest labelling intensity was observed on NV protruding from the cell surface.

The surface location of Sdp was confirmed by the abundant localization of anti-SH3b2 (85% of the particles detected). Quantification of the anti-Sdp_SH3b2 particles indicated that Sdp has significantly more abundant (*p* < 0.001) localization to NV-like structures, with 75% of particles located on NV-like structures budding off the cell surface, 5% on free NV-like structures and only 20% located elsewhere on the cell surface, (**Table S2**). These results are consistent with the TEM data where six particles were observed on cap-like structures. Similar results were obtained when Sdp detection was evaluated using anti-Sdp_SH3b6, albeit with fewer particles quantified (**Table S2**). SEM also allowed the detection of Eno3 with 96% of gold particles associated to bacterial surfaces, where 68% of the anti-Eno3 particles were associated to NV-like structures on the cell surface, 13% on free NV-like structures, and 19% were associated elsewhere on the cell wall (**Table S2**).

**Figure 7:**
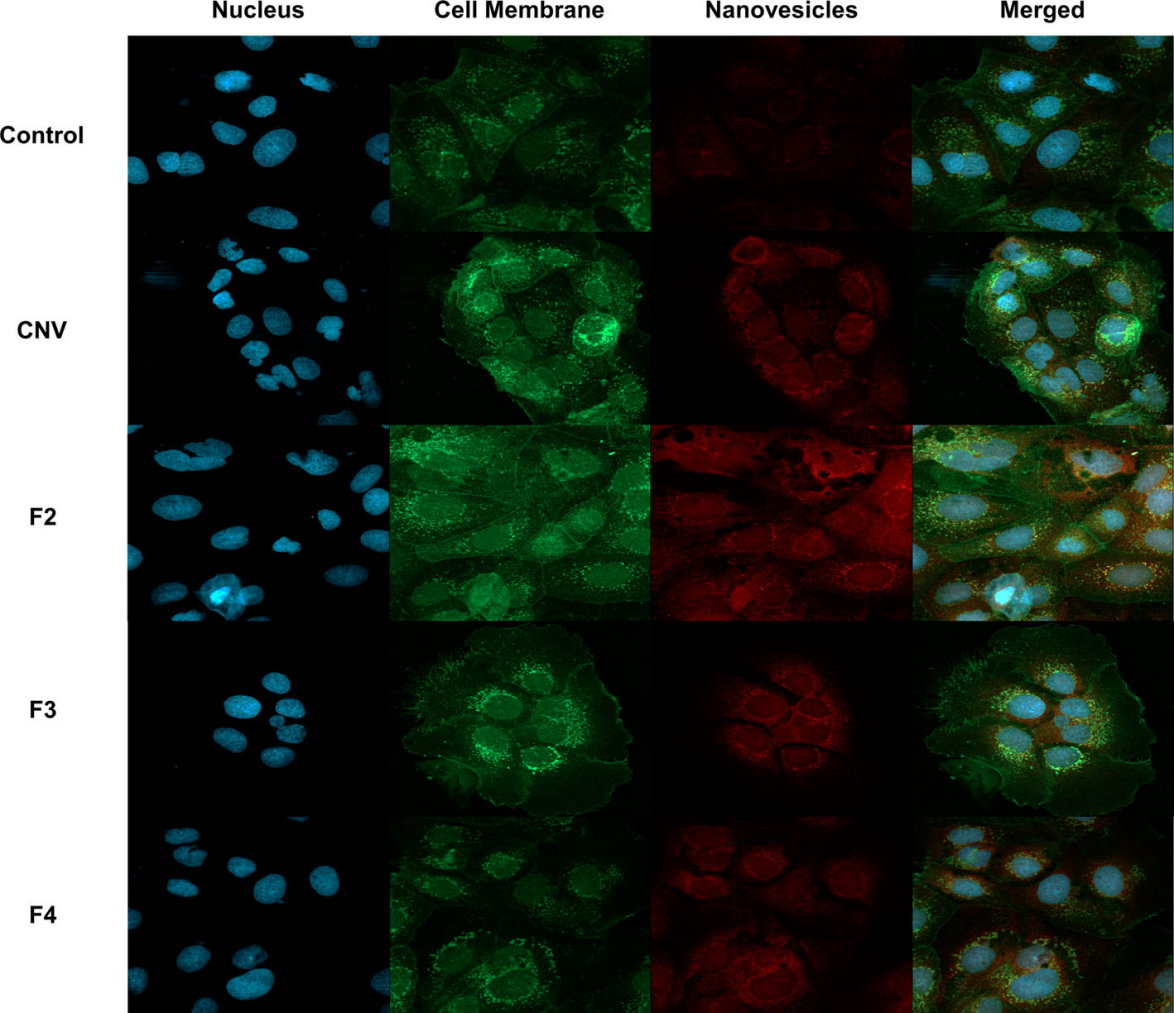
Immunofluorescence of NV associated protein Sdp_SH3b2 in Caco-2 Cells. From top to bottom, control cells, crude NV (CNV), purified NV from fractions 3-5 (F2), purified NV from fractions 6-7 (F3) and purified NV from fractions 8-9 (F4). From left to right, DAPI to stain the nucleus, Alexa 488 to stain cell membrane, Texas Red conjugate to visualize NV through anti-Sdp_SH3b2, and the merged image at a magnification of 40x.

Since technical limitations preclude immunostaining of NV, the ability of anti-Sdp to target NV was evaluated using *L. johnsonii-*derived NV internalized in Caco-2 cells, a human intestinal cell line model. Following incubation of Caco-2 cells with NV, a Texas Red conjugate against anti-Sdp_SH3b2 was used to visualize NV within the Caco-2 cells. From the images obtained, the anti-Sdp_SH3b2 labeling was observed in Caco-2 cells treated with the crude NV and purified NV fractions F2, F3 and F4 (**Figure 7**). These results indicate that Sdp_SH3b2 is associated with the NV in agreement with the immunogold assays.

### Sdp_SH3b2 and Sdp_SH3b6 Domains Can Be Recognized by Human IgA and IgG

To determine if *L. johnsonii* N6.2 NV or purified proteins can be recognized by the host, three different immunoglobulins (IgM, IgA and IgG) were quantified from plasma samples obtained from a clinical trial, where healthy adults were administered *L. johnsonii* N6.2 (27). The sample set comprises the placebo (control) group and the *L. johnsonii* N6.2 treatment group, across five different time points: baseline (T0), 2 weeks after beginning treatments (T2), week 4 (T4), week 8 end of treatment (T8), and after a four-week washout period at week 12 (T12). We adapted a method developed by Paun et al. (40) for the detection of anti-commensal antibody (ACAb), to test immunoglobulins against NV, Sdp_SH3b2, Sdp_SH3b6, Eno3 and LexA in human samples. Briefly, for each assay, NV and purified proteins (LexA, Eno3, Sdp_SH3b6, Sdp_SH3b2) were coated at 50 µg/ml. The coated plates were then incubated against the plasma samples of each subject per each timepoint (see *Materials and Methods* for details).

**Figure 8:**
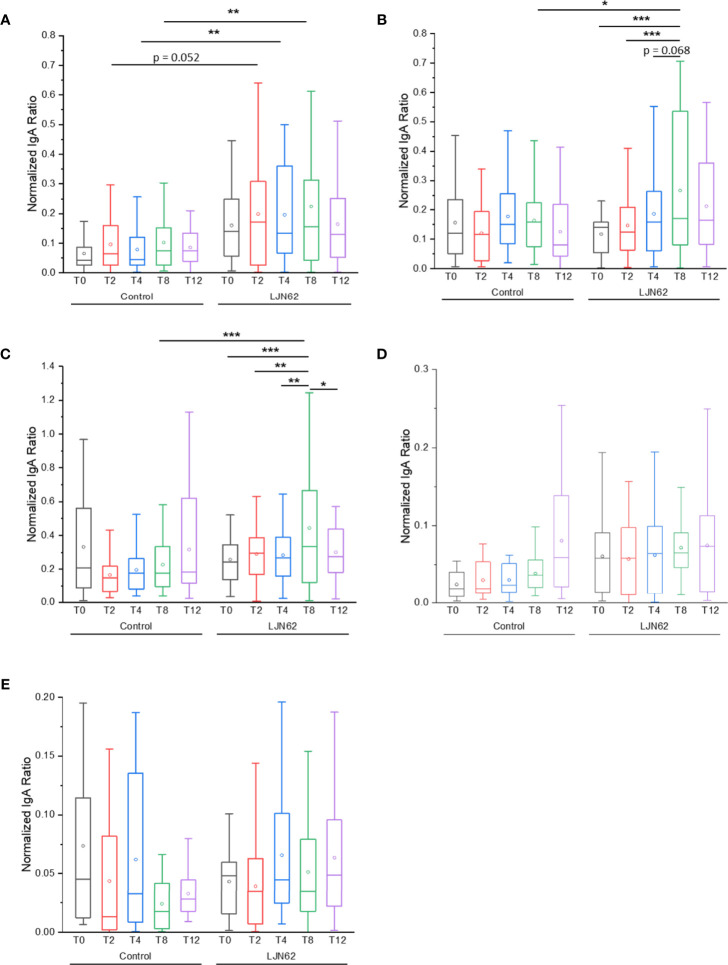
IgA ACAb generation against *L. johnsonii* N6.2 proteins and NV. Normalized IgA ratio of specific IgA generated against the antigens, NV **(A)**, Sdp_SH3b2 **(B)**, Sdp_SH3b6 **(C)**, Eno3 **(D)** and LexA **(E)**, over total plasma IgA. *p < 0.05, **p < 0.01, and ***p < 0.001.

An elevated IgA ACAb response against NV was observed at T2 in subjects that received *L. johnsonii* N6.2, however, it did not reach statistical significance (*p* = 0.052). The IgA ACAb response against NV was significantly higher at T4 (*p* = 0.008) and T8 (*p* = 0.002) in subjects that received *L. johnsonii* N6.2 (**Figure 8A**). While the IgA ACAb response against the commensal bacterial antigens indicated that NV induced a slight increase in immunogenicity over time within the treatment group, it did not reach statistical significance. The IgA ACAb response against Sdp_SH3b2 was significantly higher (*p* = 0.02) at T8 in the treatment group (**Figure 8B**). A significant increase in the level of IgA ACAb against Sdp_SH3B2 was also observed within the treatment group during the treatment period (*p* < 0.001). A similar pattern was observed for IgA ACAb response against Sdp_SH3b6 (**Figure 8C**); the IgA ACAb response against Sdp_SH3b6 was significantly higher at T8 (*p* = 0.000003) in subjects that received *L. johnsonii* N6.2. Within the treatment group, a significant increase was observed over time (*p* < 0.003). As expected, the IgA ACAb response against SH3b6 was significantly lower (*p* = 0.01) following the washout period (T8 to T12). A decrease in IgA ACAb response against NV and SH3b2 was also observed during the washout period but did not reach statistical significance. No significant differences were observed for IgA ACAb response against Eno3 or LexA, within or between treatment groups (**Figures 8D, E**).

**Figure 9:**
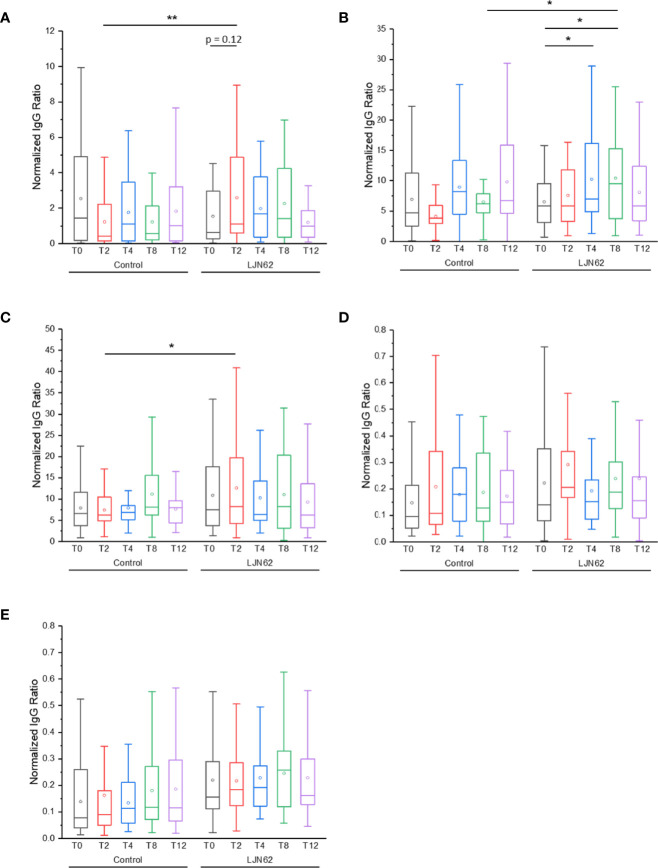
IgG ACAb generation against *L. johnsonii* N6.2 proteins and NV. Normalized IgG ratio of specific IgG generated against the antigens, NV **(A)**, Sdp_SH3b2 **(B)**, Sdp_SH3b6 **(C)**, Eno3 **(D)** and LexA **(E)**, over total plasma IgG. *p < 0.05 and **p < 0.001.

The IgG ACAb response against NV was significantly higher at T2 (*p* = 0.006) in subjects that received *L. johnsonii* N6.2 (**Figure 9A**). Within the treatment group, an increase in IgG ACAb response against NV was observed over time, however, these differences did not reach statistical significance. The IgG ACAb response against Sdp_SH3b2 was significantly higher at T8 (*p* = 0.03) in subjects that received *L. johnsonii* N6.2 (**Figure 9B**). Within the treatment group, a significant increase (*p* < 0.05) in IgG ACAb response against Sdp_SH3b2 was also observed during the treatment period (**Figure 9B**). The IgG ACAb response against Sdp_SH3b6 was significantly higher at T2 (*p* = 0.02) in subjects that received *L. johnsonii* N6.2 (**Figure 9C**), while no significant differences were observed for IgG ACAb response against Eno3 or LexA, within or between treatment groups (**Figures 9D, E**). ELISA assays were also conducted to determine the immunological response of IgM against the *L. johnsonii* N6.2 NV and proteins, however, no significant differences were observed (**Figure S6**).

## Discussion

In this study, the morphological and molecular characteristics of *L. johnsonii* N6.2 NV were elucidated and the SH3b2 and SH3b6 domains of Sdp were identified as potential biomarkers for NV. The protein and lipid composition of *L. johnsonii* N6.2 NV were found to be significantly different when compared to the CM. The lipid composition of *L. johnsonii* N6.2 NV was enriched with PG, TG and CL, whereas the lipids of the CM were enriched with DG and DGDG. The stark differences observed between the lipid composition of NV and CM suggests that NV are formed as specialized structures, rather than by random budding from the cell surface. This is further supported by the TEM and SEM imaging of NV in this study, where indications of cell lysis or cell division was not observed. In a recent article, the lipid profile of *L. plantarum* extracellular vesicles (EV) was characterized, where 67 of the 320 identified lipids were enriched in the EV, while 19 were decreased (13). Interestingly, there is no overlap between the enriched lipids identified in *L. johnsonii* NV and *L. plantarum* EVs, however, we speculate that some of the observed differences may be due to the metabolic capabilities of each species, or possibly from differences in sample preparation. In the study by Kim et al. (13), the EV is compared to total cell extracts, while in this study, we utilized exosome-depleted media during growth and purification of the NV, and we used purified cell membranes to compare to the NV.

NV lipidomic studies in *Bacillus anthracis* and Group B *Streptococcus* show a broad composition of lipids similar to their CM (19, 41). Though broadly similar in composition, the CM and NV in *Streptococcus pyogenes* and *Listeria monocytogenes* have been reported to show differences in phospholipid and fatty acid content. In *S. pyogenes*, NV are enriched in anionic phosphatidylglycerol, but cardiolipins are diminished (42), while in *L. monocytogenes*, NV are enriched in phosphatidylethanolamine, sphingolipids and triacylglycerols (8). The dissimilarities in lipid composition between NV and CM suggest that vesicle production may be promoted through lipid-enriched domains within the CM (43). This is seen in *S. aureus*, where a reduction in lipoprotein content improves membrane fluidity, resulting in increased NV production (44).

In Gram-negative bacteria, the asymmetrical distribution of phospholipids is proposed to be the major force triggering vesicle production, where an accumulation of phospholipids in the outer leaflet of the outer membrane leads to the expansion and outward bulging of the membrane (45). It has also been reported that pre-existing patches of negatively charged outer membranes are enriched with A-LPS in Gram-negative bacteria (46), and mutations that affect A-LPS biosynthesis were found to inhibit the selective sorting of cargo proteins into OMVs produced by *Porphynoomonas gingivalis* (47). In eukaryotic cells, the formation of exosomes involves lipid raft-like regions enriched in cholesterol and specific glycolipids and phospholipids, including phosphatidylcholine and sphingolipids (48). These lipid regions have been proposed to bind specific RNAs with high affinity, which are subsequently encapsulated and released as cargo within the exosomes (48). Taken together, these findings suggest that the lipid domains of bacterial cells can directly influence the cargo inside EVs.

The protein cargo in *L. johnsonii* NV was evaluated and determined to be significantly different from the CM. Proteins of intracellular localization and function were enriched in the NV, in agreement with previous reports. When comparing the NV-associated proteins identified in *L. johnsonii* N6.2 and other *Lactobacillus* species, we found some overlap between the main cytoplasmic enzymes present in the NV, however, there are numerous proteins with unique localization among species. In *L. casei* BL23 and *L. rhamnosus* (JB‐1), EVs were found to contain cytoplasmic proteins similar to *L. johnsonii* N6.2-derived NV (10, 49), while, membrane proteins were found to be more abundant in *L. plantarum*-derived EVs (20). Some proteins found at high concentrations in other *Lactobacillus-*derived vesicles, such as enolase (14), triosephosphate isomerase (14), and an adhesin protein (49) were also identified in *L. johnsonii* N6.2-derived NV. Similar observations were reported when comparing NV derived from *L. acidophilus* ATCC 53544, *L. casei* ATCC 393, and *L. reuteri* ATCC 23272, where the proteomic analysis revealed that the most abundant proteins differed among each species (50), however, some of the differences observed are likely due to the large genomic divergence and metabolic potential among these species. Taken together, these findings suggest different mechanisms of host-microbe interaction may exist within commensal bacteria from the same genus, however, further studies are needed to establish genomic and proteomic correlations to fully understand these differences.

Two proteins (Eno3 and Sdp) were purified and utilized in localization and immunogenicity studies. Enolase is a multifunctional protein that has recently been identified in several bacterial-derived EVs and OMVs. While the enzymatic activity of enolase is most commonly associated with glycolysis and gluconeogenesis, active forms of enolase have also been found to play a role in virulence and other activities unrelated to central metabolism, as seen in *Mycobacterium tuberculosis* (51) and in *Borrelia burgdorferi*, where an enolase was identified as a plasminogen receptor released by OMVs (52). In the fungal pathogen *Aspergillus fumigatus*, enolase also acts as a virulence factor in recruiting human regulators (53), and in *Trichomonas vaginalis*, enolase plays a role in virulence by activating plasminogen to plasmin (54). Interestingly, some commensal bacteria, including *L. johnsonii, L. crispatus* and *L. plantarum*, also encode enolases that can bind plasminogen and activate plasmin (55), among other moonlighting activities. A surface-associated protein homologous to enolase (Eno3) from *L. gasseri* ATC 3323 was found to inhibit the adherence of *Neisseria gonorrhoeae* to epithelial cells (55). Similar to *L. gasseri* ATCC 33323, *L. johnsonii* N6.2 also has three enolase genes, and our results support the surface localization of Eno3 in *L. johnsonii* N6.2. Using immunogold staining and TEM, we observed that Eno3 is associated with membranes and present in the cytoplasm, and SEM showed association of Eno3 on the cell surface. While these results are consistent with observations of Eno3 from *L. gasseri*, future studies are needed to determine if *L. johnsonii* N6.2 Eno3 has a similar role in pathogen exclusion.

Immunogold staining of the Sdp_SH3b2 and Sdp_SH3b6 domains in *L. johnsonii* N6.2 support the membrane localization and association of these domains on the surface of NV. While the function of bacterial SH3b domains is not well known, they are proposed to be cell wall binding domains in prokaryotes (56, 57). SH3b domains have been identified on diverse proteins in conjunction with a variety of domains, including *N-*acetylmuramyl-L-alanine amidase, peptidase U20 family, peptidase M23 family, lysins (LysM), kinases and lysozymes (58). SH3b domains were recently reported to have two binding sites, in which one recognizes the pentaglycine crossbridge and the other recognizes the peptide stem of the cell wall (59). In *Staphylococcus simulans*, a lysostaphin SH3b domain binds to the pentaglycine interpeptide bridges of *S. aureus* peptidoglycan (60), while the SH3b domain of a phage endolysin was able to bind three types of staphylococcal peptidoglycan and *Streptococcus uberis* in absence of pentaglycine (57). While the primary biological function of Sdp may be related to the linkage of pentaglycine bridges, to bind to the cell wall, it is also thought to play a role in other moonlighting functions. A study regarding the diphtheria toxin repressor reported that the C-terminal domain resembled the folding structure of SH3 domains in eukaryotes (61). They presented structural evidence that the SH3 domain binds to a proline-rich segment linking the N- and C-terminal domains of the repressor. This binding interaction is thought to activate the repressor and decrease toxin expression (61). Structural and biophysical analysis also indicated the proline-rich peptide may function as a switch to modulate the activation of repressor activity (62). While the potential regulatory role of Sdp was not examined in this study, these finding support further investigation into the moonlighting activity of Sdp in *L. johnsonii*. Overall, it was observed that Sdp_SH3b2 and Sdp_SH3b6 had more immunogold particles located on NV-like structures on the cell surface, or surrounding the cell when compared to Eno3, supporting its extracellular display.

To determine if proteins localized on the surface of NV could be used as biomarkers of *in vivo* production, we examined the potential for NV proteins to generate an immune response in human subjects. To this end, an ELISA assay was developed using purified NV, Sdp_SH3b2, Sdp_SH3b6, Eno3 and LexA proteins, to evaluate the host ACAb response to *L. johnsonii* N6.2 administration. The Sdp_SH3b2 and Sdp_SH3b6 domains generated the strongest IgA ACAb response, followed by whole NV, while no ACAb response was observed with Eno3 or LexA. In our previous study, we found that the total IgA concentration increased over time in the group that received *L. johnsonii* N6.2 (27). Here, we were able to show that the increase in IgA resulted from a specific response to components of the *L. johnsonii* N6.2 NV. In addition, a similar pattern was observed with IgG. Previous studies had used whole bacterial cells to study the host response to commensal microorganisms. Paun et al. (40), evaluated the titers and isotypes of ACAb to determine if patterns of ACAb responses are associated with T1D. The sera from subjects with recent-onset T1D and healthy controls exhibited differences in the IgA ACAb response against *L. acidophilus*, and the ACAb responses to intestinal microbes were found to be associated with the *HLA-DR* genotype, islet autoantibody and future diagnosis of T1D (40). A similar study found that commensal bacteria (Proteobacteria-rich) induce the serum levels of IgA, providing a protective effect against polymicrobial sepsis (63). Here, were able to show that the increased IgA production previously observed in subjects administered *L. johnsonii* N6.2 is due to the sensing of specific components present in the NV. In agreement with these findings we observed a decrease in the level of IgA recognizing NV, Sdp_SH3b2 and Sdp_SH3b6 after the washout period, which suggests continuous administration of the bacteria may be needed to confer benefits to the host. A limitation of the immunogenicity data obtained against NVs or purified proteins is that the clinical trial was designed to evaluate the immunological response to whole bacteria, in contrast to purified components. As such, further studies in animal models are needed to confirm these observations.

The production of NV by beneficial microorganisms offers an alternative mechanism of host microbe interactions, however, many questions remain unanswered. Among them are how these structures are formed and released through the peptidoglycan layer (and possibly S-layer), how these structures interact with host cells (either epithelial or immune cells), and do NV only exert an effect on the host locally or can they potentially “travel” to distant locations. While scarce information is available on OMVs entering the bloodstream from intestinal epithelial cells (64, 65), the beneficial effects of administering pure EVs have been reported. There are no previously reported biomarkers for tracking the movement of vesicles in the host, however, in this work we characterized NV from *L. johnsonii* N6.2 and identified Sdp as a biomarker for NV. We were able to further determine that subjects consuming *L. johnsonii* N6.2 are able to generate IgA and IgG against the Sdp_SH3b domains, which were found to be enriched in NV. Future studies will be directed to determine if NV are sensed by antigen-presenting cells at the intestinal interphase and/or following systemic distribution upon entering the bloodstream.

## Data Availability Statement

The datasets presented in this study can be found in online repositories. The names of the repository/repositories and accession number(s) can be found below: http://www.ebi.ac.uk/pride/archive/projects/PXD027785.

## Ethics Statement

The studies involving human participants were reviewed and approved by Institutional Review Board at the University of Florida. The patients/participants provided their written informed consent to participate in this study.

## Author Contributions

NH and DS performed the research. NH, CG, DS, CG, and GL wrote the manuscript and conceived the study. All authors contributed to the article and approved the submitted version.

## Funding

This study was partially funded by the National Center for Advancing Translational Sciences of the National Institutes of Health under University of Florida Clinical and Translational Science Awards TL1TR001428 and UL1TR001427 and the National Institute of Diabetes and Digestive and Kidney Diseases of the National Institutes of Health under award number 1R01DK121130. The content is solely the responsibility of the authors and does not necessarily represent the official views of the National Institutes of Health.

## Author Disclaimer

The content is solely the responsibility of the authors and does not necessarily represent the official views of the National Institutes of Health.

## Conflict of Interest

GL holds U.S. Patent No. 9,474,773.

The remaining authors declare that the research was conducted in the absence of any commercial or financial relationships that could be construed as a potential conflict of interest.

## Publisher’s Note

All claims expressed in this article are solely those of the authors and do not necessarily represent those of their affiliated organizations, or those of the publisher, the editors and the reviewers. Any product that may be evaluated in this article, or claim that may be made by its manufacturer, is not guaranteed or endorsed by the publisher.

## Supplementary Material

Supplementary Figure 1 SDS-PAGE Gel of *L. johnsonii* N6.2 protein composition of different cellular components. From left to right, the gel shows the molecular weight ladder (MW), total extract (TE), intracellular extract (IC), cell membrane (CM) and nanovesicles (NV).

Supplementary Figure 2 Graphical illustration of the conserved domains found in T285_RS00825, and SDS-PAGE gel of purified proteins. **(A)** Conserved domains found in T285_RS00825 (top row) and selected domains for protein purification (bottom row). The amino acid sequence is 1 to 935 amino acids. Glyco_25 – Glycosyl hydrolases family 25; Lysozyme M1; muramidase and SH3b – Bacterial SH3 Domain Homologs. **(B)** Multiple alignment of the six Sdp_SH3b domains found in the T285_RS00825 protein. **(C)** From left to right: molecular weight ladder, SH3b2 domain, SH3b6 domain, Enolase 3, and LexA.

Supplementary Figure 3 Phylogenetic tree of SMART domains, Glyco_25 and Sdp_SH3b, in relation to T285_RS00825. Royal blue clade = *Lactobacillus;* Cyan clade = *Pediococcus*; Gray-blue clade = *Enterococcus*; Navy blue clade = *Streptococcus*; Green clade = Bacillales; Orange clade = *Clostridium*; Black clade = Anerolineaceae; Yellow clade = *Aciduliprofundum*; Purple clade = *Caudovirales.*

Supplementary Figure 4 LexA expression under aerobic conditions. A representative western blot of LexA under static conditions, aerobic conditions and the purified LexA protein.

Supplementary Figure 5 Optiprep purification of crude NV by ultracentrifugation. **(A)** Western blot of the specificity of the Sdp_SH3b2 domain in protein precipitated Optiprep fractions: 1 through 12 (from top to bottom of the tube) and their respective densities. **(B)** Nanosight size and concentration of crude NV (solid line), fractions 1-2 (F1; long dashed lines), fractions 3-5 (F2; dots), fractions 6-7 (F3; short dashed lines) and fractions 8-9 (F4; dot and dashed line). **(C)** From left to right, western blots showing specificity of Sdp_SH3b2, Sdp_SH3b6, Eno3 and LexA in the fractions 1-2 (F1), fractions 3-5 (F2), fractions 6-7 (F3), fractions 8-9 (F4), the crude NV (CNV) and the respective purified protein.

Supplementary Figure 6 IgM ACAb generation against *L. johnsonii* N6.2 proteins and NV. Normalized IgM ratio of specific IgM generated against the antigens, NV **(A)**, Sdp_SH3b2 **(B)**, Sdp_SH3b6 **(C)**, Eno3 **(D)** and LexA **(E)**, over total plasma IgM.
