# Supplementary figures and images for: Identification of Biomarkers for Systemic Distribution of Nanovesicles From Lactobacillus johnsonii N6.2
Source: Front Immunol. 2021 Aug 31;12:723433. doi: 10.3389/fimmu.2021.723433 (PMC8438180; doi:10.3389/fimmu.2021.723433)

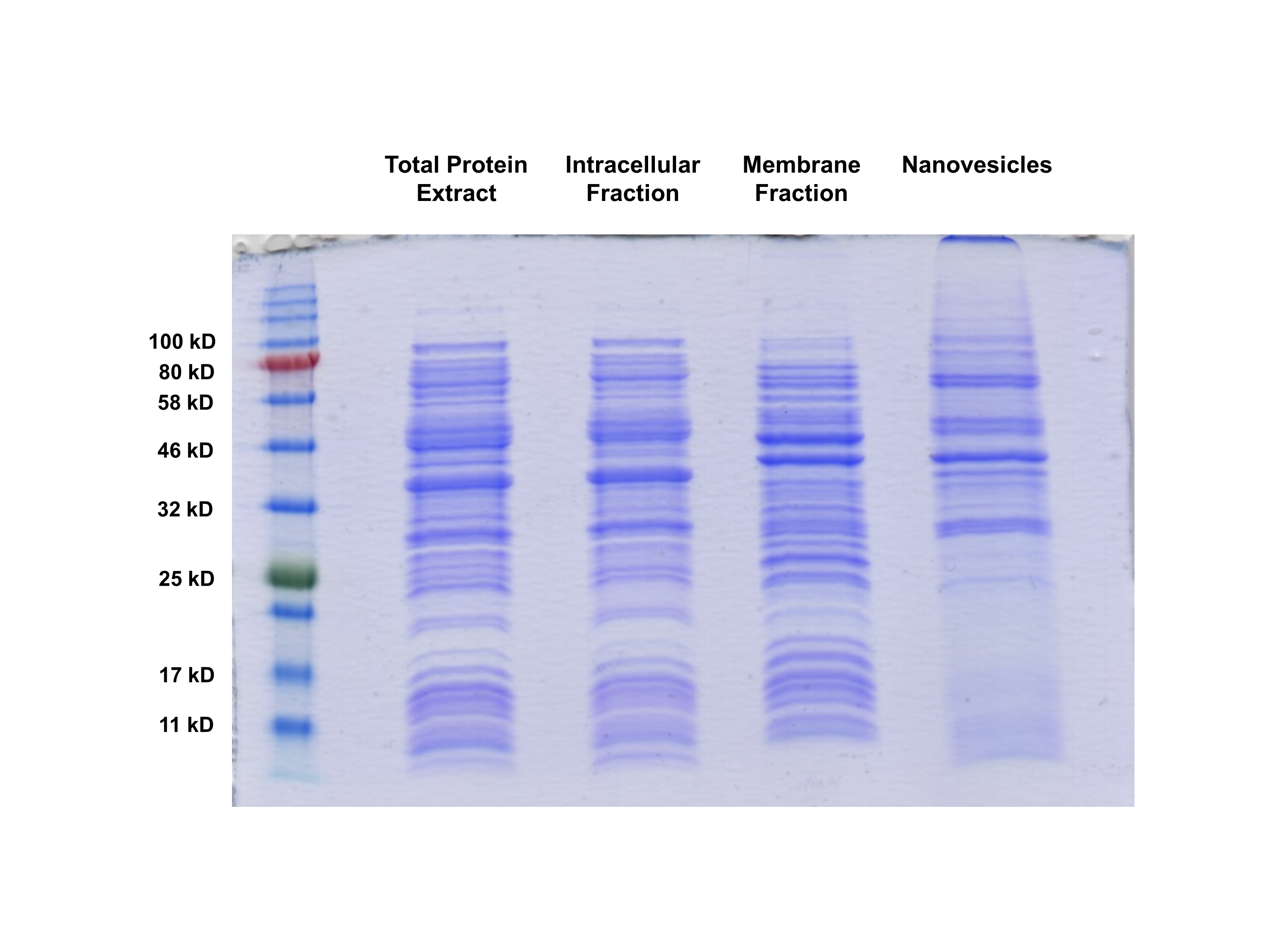

Supplement: Supplementary Figure 1 — SDS-PAGE Gel of L. johnsonii N6.2 protein composition of different cellular components. From left to right, the gel shows the molecular weight ladder (MW), total extract (TE), intracellular extract (IC), cell membrane (CM) and nanovesicles (NV). [file Image_1.tif]

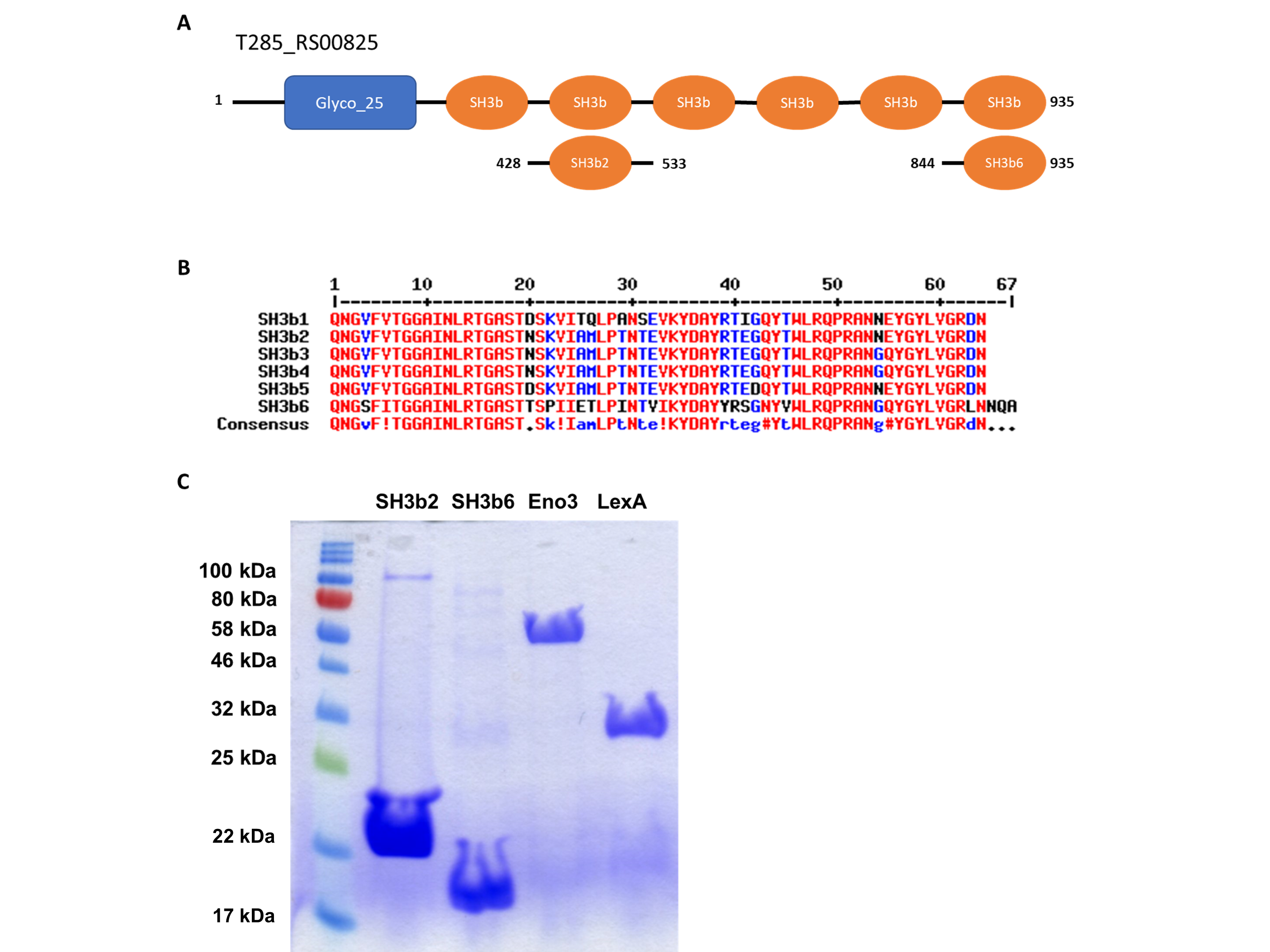

Supplement: Supplementary Figure 2 — Graphical illustration of the conserved domains found in T285_RS00825, and SDS-PAGE gel of purified proteins. (A) Conserved domains found in T285_RS00825 (top row) and selected domains for protein purification (bottom row). The amino acid sequence is 1 to 935 amino acids. Glyco_25 – Glycosyl hydrolases family 25; Lysozyme M1; muramidase and SH3b – Bacterial SH3 Domain Homologs. (B) Multiple alignment of the six Sdp_SH3b domains found in the T285_RS00825 protein. (C) From left to right: molecular weight ladder, SH3b2 domain, SH3b6 domain, Enolase 3, and LexA. [file Image_2.tif]

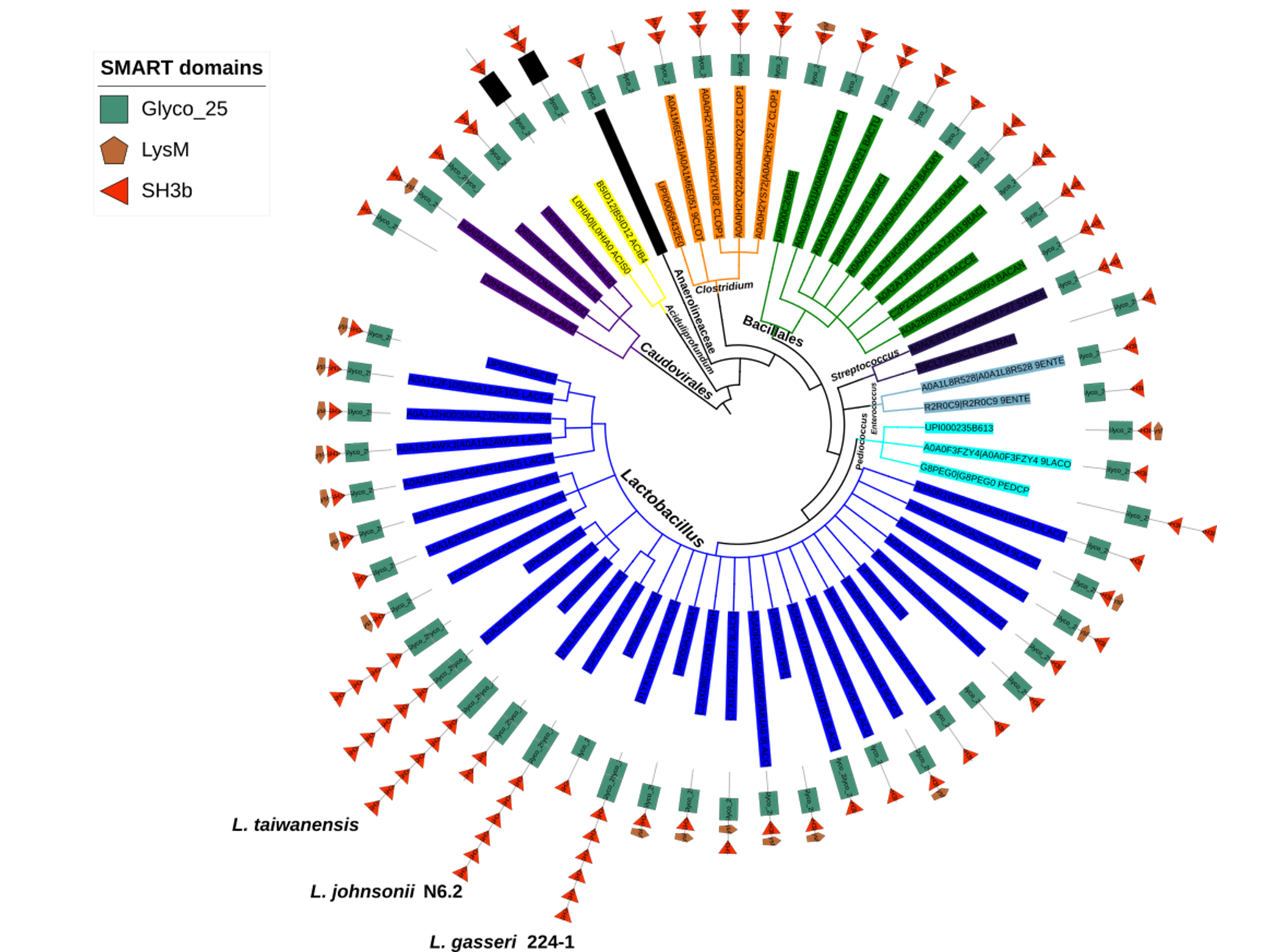

Supplement: Supplementary Figure 3 — Phylogenetic tree of SMART domains, Glyco_25 and Sdp_SH3b, in relation to T285_RS00825. Royal blue clade = Lactobacillus; Cyan clade = Pediococcus; Gray-blue clade = Enterococcus; Navy blue clade = Streptococcus; Green clade = Bacillales; Orange clade = Clostridium; Black clade = Anerolineaceae; Yellow clade = Aciduliprofundum; Purple clade = Caudovirales. [file Image_3.tif]

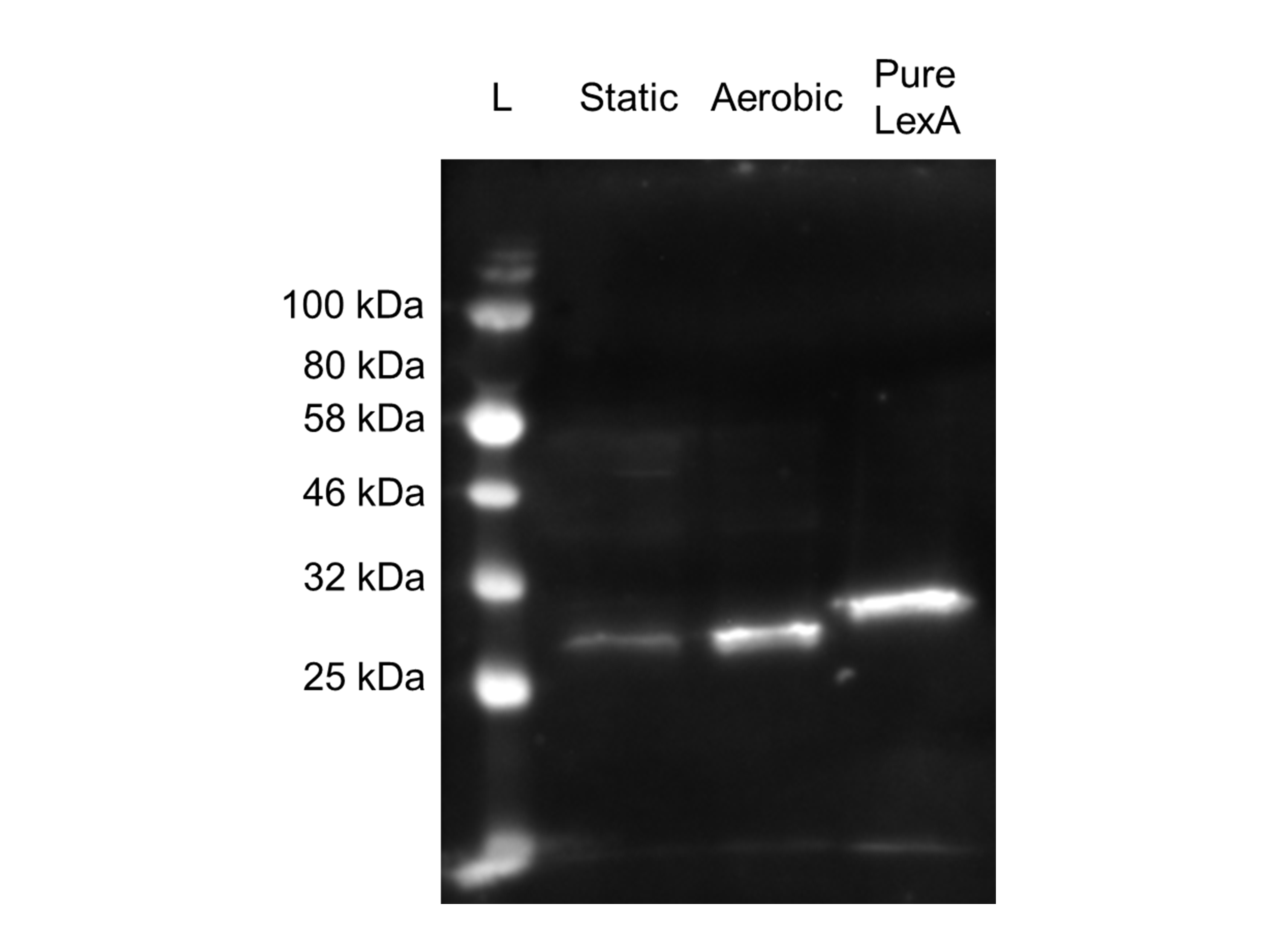

Supplement: Supplementary Figure 4 — LexA expression under aerobic conditions. A representative western blot of LexA under static conditions, aerobic conditions and the purified LexA protein. [file Image_4.tif]

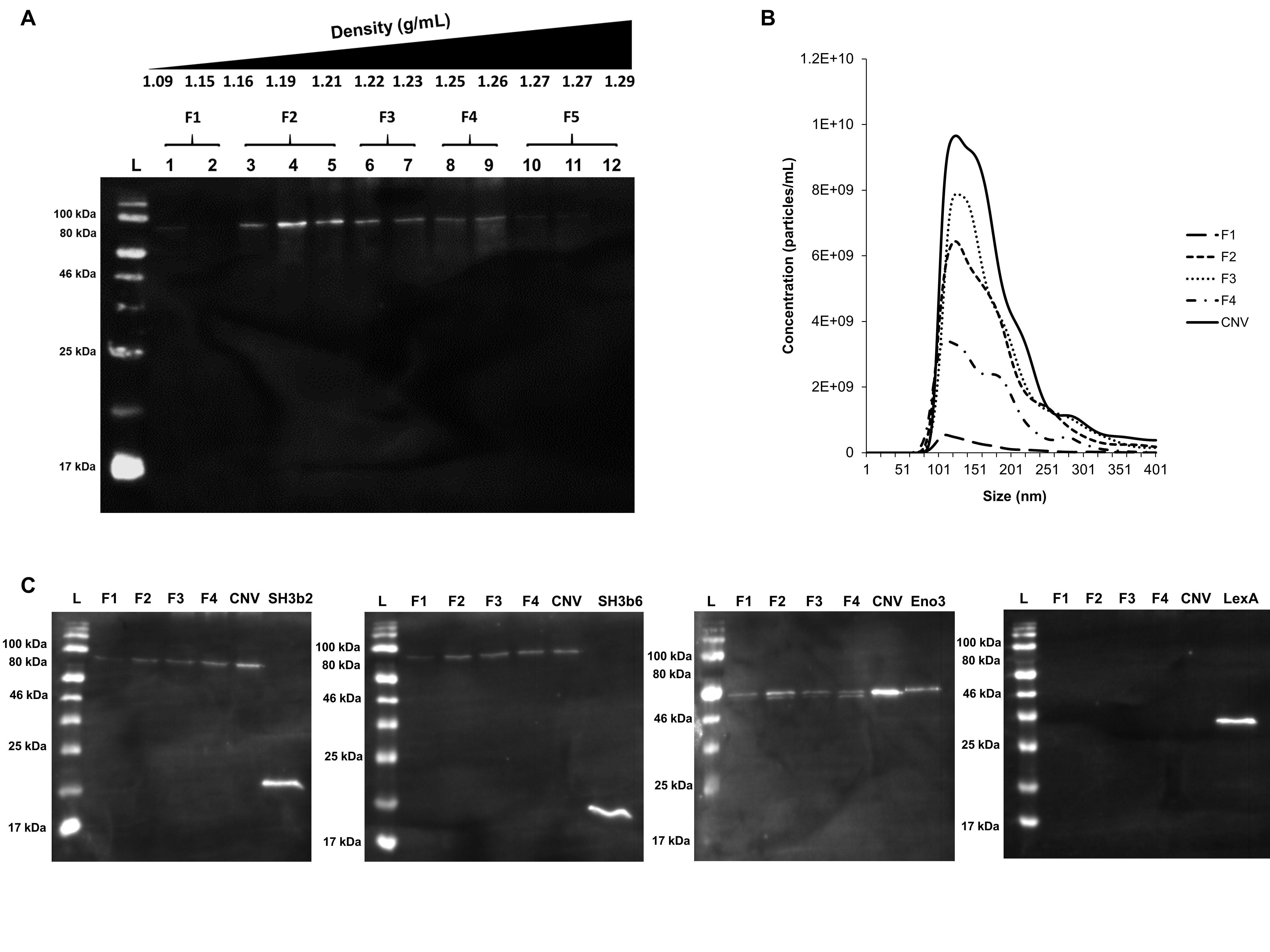

Supplement: Supplementary Figure 5 — Optiprep purification of crude NV by ultracentrifugation. (A) Western blot of the specificity of the Sdp_SH3b2 domain in protein precipitated Optiprep fractions: 1 through 12 (from top to bottom of the tube) and their respective densities. (B) Nanosight size and concentration of crude NV (solid line), fractions 1-2 (F1; long dashed lines), fractions 3-5 (F2; dots), fractions 6-7 (F3; short dashed lines) and fractions 8-9 (F4; dot and dashed line). (C) From left to right, western blots showing specificity of Sdp_SH3b2, Sdp_SH3b6, Eno3 and LexA in the fractions 1-2 (F1), fractions 3-5 (F2), fractions 6-7 (F3), fractions 8-9 (F4), the crude NV (CNV) and the respective purified protein. [file Image_5.tif]

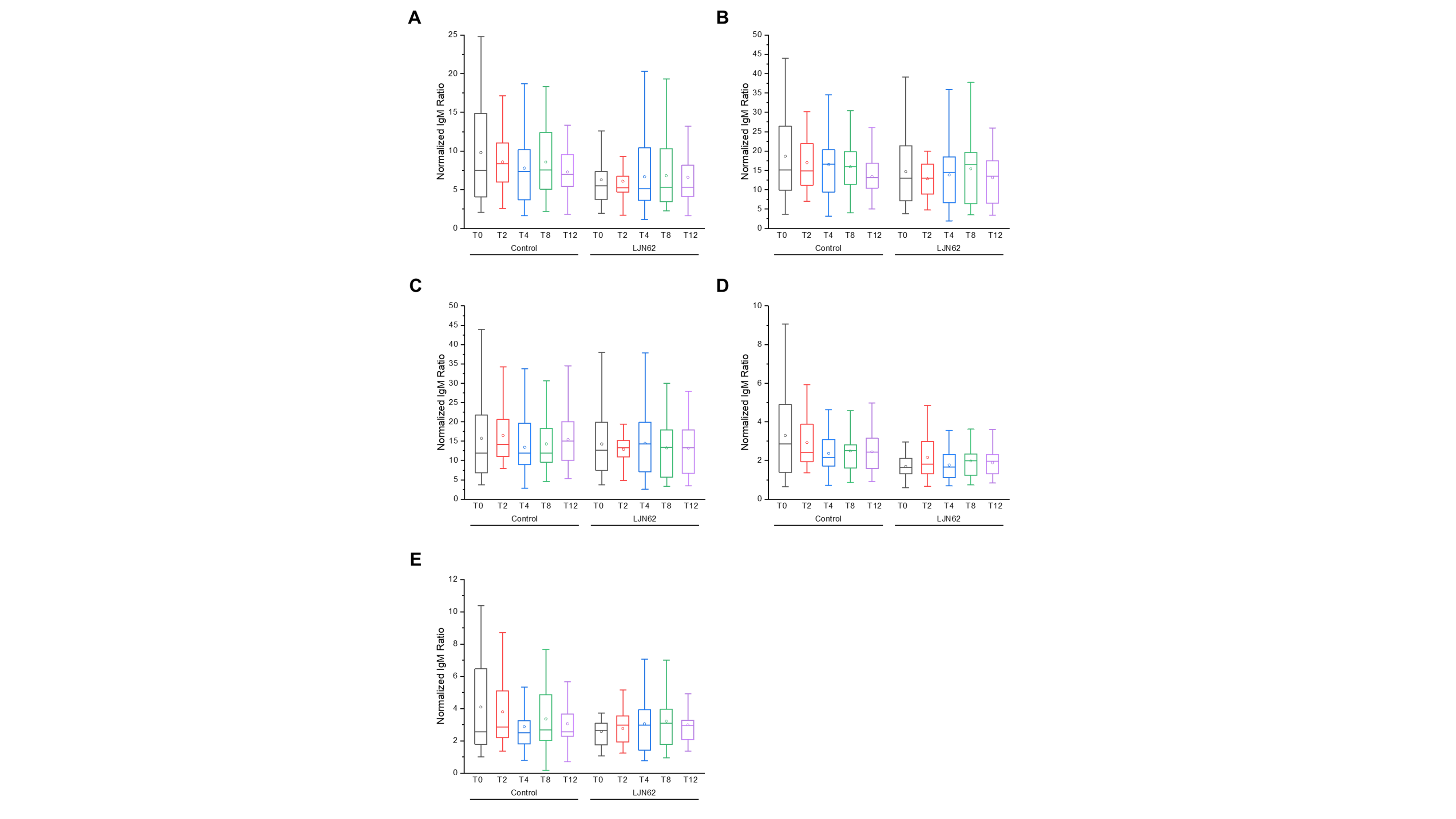

Supplement: Supplementary Figure 6 — IgM ACAb generation against L. johnsonii N6.2 proteins and NV. Normalized IgM ratio of specific IgM generated against the antigens, NV (A), Sdp_SH3b2 (B), Sdp_SH3b6 (C), Eno3 (D) and LexA (E), over total plasma IgM. [file Image_6.tif]
