# Supplementary material for: Identification of Biomarkers for Systemic Distribution of Nanovesicles From Lactobacillus johnsonii N6.2
Source: Front Immunol. 2021 Aug 31;12:723433. doi: 10.3389/fimmu.2021.723433 (PMC8438180; doi:10.3389/fimmu.2021.723433)
Supplement: Supplementary file 7 [file Table_1.docx]

Supplementary Material

**Supplementary Table 1.** Quantification of the Immunogold particles against selected proteins by TEM.

|  | SH3b2 | SH3b6 | Enolase | LexA |
| --- | --- | --- | --- | --- |
| **Bacterial Particles** | **94% (n=855)** | **48% (n=212)** | **99% (n=7613)** | **23% (n=52)** |
| Cell Wall | 3% (n=25) | 13% (n=28) | 4% (n=273) | 12% (n=6) |
| Cell Membrane | 25% (n=217) | 30% (n=64) | 16% (n=1245) | 40% (n=21) |
| Cytoplasm | 71% (n=607) | 55% (n=117) | 80% (n=6095) | 48% (n=25) |
| Cap Structure | 1% (n=6) | 1% (n=3) | - | - |
| **Free Particles** | **6% (n=52)** | **52 % (n=227)** | **1% (n=74)** | **77% (n=172)** |
| Total Particles | n=907 | n=439 | n=7687 | n=224 |

**Supplementary Table 2.** Quantification of the Immunogold particles against selected proteins by SEM.

|  | **SH3b2** | **SH3b6** | **Enolase** |
| --- | --- | --- | --- |
| **Bacterial Particles** | **85% (n=949)** | **94% (n=289)** | **96% (n=418)** |
| Cell Wall | 20% (n=195) | 16% (n=47) | 19% (n=80) |
| NV-like Structures from Cell Wall | 75% (n=711) | 81% (n=235) | 68% (n=282) |
| Free NV-like Structures | 5% (n=43) | 3% (n=7) | 13% (n=56) |
| **Free Particles** | **15% (n=163)** | **6% (n=18)** | **4% (n=19)** |
| Total Particles | n=1112 | n=307 | n=437 |
